# Supplementary material for: m6A demethylase ALKBH5 promotes tumor cell proliferation by destabilizing IGF2BPs target genes and worsens the prognosis of patients with non-small-cell lung cancer
Source: Cancer Gene Ther. 2022 Mar 22;29(10):1355–72. doi: 10.1038/s41417-022-00451-8 (PMC9576599; doi:10.1038/s41417-022-00451-8)
Supplement: Supplementary file 2 — Supplementary Table Tsuchiya [file 41417_2022_451_MOESM2_ESM.docx]

**Table S1. Primer list for qPCR**

| primer name | number |  | sequence |
| --- | --- | --- | --- |
| GAPDH |  | Forward | AGGTGAAGGTCGGAGTCAAC |
|  |  | Reverse | TTGAGGTCAATGAAGGGGTC |
| ALKBH5 |  | Forward | CGGCGAAGGCTACACTTACG |
|  |  | Reverse | CCACCAGCTTTTGGATCACCA |
| FTO |  | Forward | CTTCACCAAGGAGACTGCTATTTC |
|  |  | Reverse | CAAGGTTCCTGTTGAGCACTCTG |
| E2F1 |  | Forward | TCCCTGAGCTGTTCTTCTGC |
|  |  | Reverse | AGTCTGTCTCCCTCCCTCAC |
| CDKN1A | primer1 | Forward | GCCGAAGTCAGTTCCTTGTG |
|  |  | Reverse | CATGGGTTCTGACGGACATC |
|  | primer2 | Forward | TCTCAGGGTCGAAAACGGC |
|  |  | Reverse | CGGATTAGGGCTTCCTCTTGG |
|  | primer3 | Forward | GACCCCAAACACCTTCCAGC |
|  |  | Reverse | TAGGTGGAGAAACGGGAACC |
| CCNG2 |  | Forward | TGAACACTGCACCTGTCTCTG |
|  |  | Reverse | TGCTACTCTATATTGCCCAGTTC |
| GADD45A |  | Forward | CTACACTGATGCAAGGATTACAGA |
|  |  | Reverse | TCCTTCTTCATTTTCACCTCTTTCC |
| AGR2 |  | Forward | ACACAAAGGACTCTCGACCC |
|  |  | Reverse | CATCAAGGGTTTGTTGCTTGTC |
| CASP14 |  | Forward | AAGCCAGCCAAAACTTAGCAC |
|  |  | Reverse | AATGCACTCTTAGGTCTCTTCGG |
| TIMP3 | primer1 | Forward | GCACGGCAACTTTGGAGAG |
|  |  | Reverse | AGCCAAGGGGTCATTGC |
|  | primer2 | Forward | CAATGACCCCTTGGCTCGG |
|  |  | Reverse | AGCATGTGCACGCCTCG |
|  | primer3 | Forward | GCCCTTCTCCTCCAATACATAA |
|  |  | Reverse | GAGTCTATCTGCTTGCTGCCT |
| PMAIP1 |  | Forward | CCAAACTCTTCTGCTCAGGAACC |
|  |  | Reverse | TGATGAAACGTGCACCTCCTG |
| ELF3 |  | Forward | GCCAGATACCTCAGCGCTAC |
|  |  | Reverse | CATGAGGCTACCGGAGTGG |
| AKAP12 |  | Forward | CAGCCACCGAAATGTTGACG |
|  |  | Reverse | TTCTCCGTTTCCTGTCCAGC |
| MFAP5 | primer1 | Forward | AGAGAGACACATTCAGCAGCC |
|  |  | Reverse | GGTCCCAAGAGCGACATATTG |
|  | primer2 | Forward | AATGTGGATTTGCAGAGACC |
|  |  | Reverse | CCTCCTTCCTCTCACCCATA |
|  | primer3 | Forward | TGGGGCTAAAGCATGAGGAC |
|  |  | Reverse | ACTTCCCGACTAGACAACTGC |
| IGF2BP1 |  | Forward | ATAGCTCCTTTATGCAGGCTC |
|  |  | Reverse | CGGGTGGTGCAATCTTGATG |
| IGF2BP2 |  | Forward | AAACATCCCTCCTCACCTGC |
|  |  | Reverse | GTGTCTGTGTTGACTTGTTCC |
| IGF2BP3 |  | Forward | CTTCTATGCTTGCCAGGTTGC |
|  |  | Reverse | GAGCCTTCTGTTGTTGGTGC |
| RPL32 |  | Forward | TGTGAAGCCCAAGATCGTCA |
|  |  | Reverse | GTCAATGCCTCTGGGTTTCC |

**Table S2. Clinical characteristics based on ALKBH5 and FTO expression in non-small cell lung cancer**

|  |  | **ALKBH5** | |  | **FTO** | |  |
| --- | --- | --- | --- | --- | --- | --- | --- |
|  | Total | low | high |  | low | high |  |
|  | N = 627 | N = 315 | N = 312 | p-value | N = 313 | N = 314 | p-value |
| Age |  |  |  |  |  |  |  |
| Median (range) | 68 (23–88) | 68 (23–88) | 69 (39–85) | 0.074 | 69 (23–88) | 68 (34–85) | 0.356 |
| Sex, n (%) |  |  |  |  |  |  |  |
| Male | 430 (68.6) | 214 (67.9) | 216 (69.2) | 0.732 | 223 (71.2) | 207 (65.9) | 0.169 |
| Female | 197 (31.4) | 101 (32.1) | 96 (30.8) |  | 90 (28.8) | 107 (34.1) |  |
| Smoking history, n (%) |  |  |  |  |  |  |  |
| Never | 184 (29.3) | 100 (31.7) | 84 (26.9) | 0.389 | 85 (27.2) | 99 (31.5) | 0.389 |
| Ever | 432 (68.9) | 209 (66.3) | 223 (71.5) |  | 221 (70.6) | 211 (67.2) |  |
| Unkown | 11 (1.8) | 6 (1.9) | 5 (1.6) |  | 7 (2.2) | 4 (1.3) |  |
| Histology, n (%) |  |  |  |  |  |  |  |
| Adenocarcinoma | 413 (65.9) | 205 (65.1) | 208 (66.7) | 0.151 | 204 (65.2) | 209 (66.6) | 0.184 |
| Squamous cell carcinoma | 170 (27.1) | 93 (29.5) | 77 (24.7) |  | 92 (29.4) | 78 (24.8) |  |
| Others | 44 (7.0) | 17 (5.4) | 27 (8.7) |  | 17 (5.4) | 27 (8.6) |  |
| Tumor status, n (%) |  |  |  |  |  |  |  |
| 1 | 255 (40.7) | 138 (43.8) | 117 (37.5) | 0.149 | 145 (46.3) | 110 (35.0) | 0.002 |
| 2 | 273 (43.5) | 123 (39.0) | 150 (48.1) |  | 114 (36.4) | 159 (50.6) |  |
| 3 | 62 (9.9) | 33 (10.5) | 29 (9.3) |  | 31 (9.9) | 31 (9.9) |  |
| 4 | 37 (5.9) | 21 (6.7) | 16 (5.1) |  | 23 (7.3) | 14 (4.5) |  |
| Node metastasis, n (%) |  |  |  |  |  |  |  |
| 0 | 467 (74.5) | 252 (80.0) | 215 (68.9) | 0.003 | 245 (78.3) | 222 (70.7) | 0.004 |
| 1 | 69 (11.0) | 28 (8.9) | 41 (13.1) |  | 27 (8.6) | 42 (13.4) |  |
| 2 | 84 (13.4) | 30 (9.5) | 54 (17.3) |  | 38 (12.1) | 46 (14.6) |  |
| 3 | 7 (1.1) | 5 (1.6) | 2 (0.6) |  | 3 (1.0) | 4 (1.3) |  |
| Pathological stage, n (%) |  |  |  |  |  |  |  |
| 1 | 395 (63.0) | 213 (67.6) | 182 (58.3) | 0.055 | 211 (67.4) | 184 (58.6) | 0.034 |
| 2 | 111 (17.7) | 49 (15.6) | 62 (19.9) |  | 44 (14.1) | 67 (21.3) |  |
| 3 | 121 (19.3) | 53 (16.8) | 68 (21.8) |  | 58 (18.5) | 63 (20.1) |  |
| Chemotherapy, n (%) |  |  |  |  |  |  |  |
| + | 258 (41.1) | 102 (32.4) | 156 (50.0) | <0.001 | 77 (24.6) | 181 (57.6) | <0.001 |
| - | 369 (58.9) | 213 (67.6) | 156 (50.0) |  | 236 (75.4) | 133 (42.4) |  |
| EGFR mutation with IHC, n (%) |  |  |  |  |  |  |  |
| Wild type | 500 (79.7) | 266 (84.4) | 234 (75.0) | 0.004 | 269 (85.9) | 231 (73.6) | <0.001 |
| Mutant | 127 (20.3) | 49 (15.6) | 78 (25.0) |  | 44 (14.1) | 83 (26.4) |  |

Differences between experimental groups were assessed using Mann–Whitney *U*-test for continuous variables or Fisher’s exact test for categorical data. Data represent median (range) or number (n) (%). IHC; immunohistochemistry

**Table S3. Multivariate Cox hazards models of survivals in all patients with non-small cell lung cancer**

| *Variable* | Per unit for HR | Univariate analysis | | |  | Multivariate analysis | | |
| --- | --- | --- | --- | --- | --- | --- | --- | --- |
|  |  | HR | 95% CI | *p*-value |  | HR | 95% CI | *p*-value |
| Age | 1-year | 1.023 | 1.007–1.040 | 0.005 |  | 1.039 | 1.019–1.060 | <0.001 |
| Sex | Male/Female | 0.406 | 0.275–1.446 | <0.001 |  | 1.267 | 0.686–2.331 | 0.447 |
| Smoking status | Ever/Never | 3.101 | 2.009–4.786 | <0.001 |  | 3.024 | 1.510-6.053 | <0.001 |
| Histology | Ad to Sq | 2.008 | 1.446–2.790 | <0.001 |  | 1.116 | 0.766–1.625 | 0.568 |
| Stage | 1-stage | 2.127 | 1.795–2.521 | <0.001 |  | 2.307 | 1.943–2.739 | <0.001 |
| EGFR | Mutant/wild | 0.589 | 0.379–0.914 | 0.018 |  |  |  |  |
| chemotherapy | +/− | 1.054 | 0.776–1.431 | 0.736 |  |  |  |  |
| ALKBH5 | High to Low | 1.675 | 1.230–2.521 | 0.001 |  | 1.468 | 1.039–2.073 | 0.029 |
| FTO | High to Low | 1.222 | 0.900–1.659 | 0.2 |  |  |  |  |

Prediction of mortality of patients with non–small-lung cancer. The univariate and multivariate Cox proportional hazards models were applied to generate the hazard ratios (HRs) of death. Multivariate analysis was adjusted by age, sex, smoking status, histology, stage, and ALKBH5. HR; hazard ratio, CI; confdence interval, Ad; adenocarcinoma, Sq; squamous cell carcinoma

**Table S4. Differentially upregulated genes in expression microarray analysis**

|  | siALKBH5 #1 vs siNC (n=3) | | | | |  | siALKBH5 #3 vs siNC (n=3) | | | | |
| --- | --- | --- | --- | --- | --- | --- | --- | --- | --- | --- | --- |
| Gene Symbol | Average value of siALKBH5 | Average value of siNC | Ratio {siALKBH5/siNC} | Log2Ratio {siALKBH5/siNC} | *P*-value |  | Average value of siALKBH5 | Average value of siNC | Ratio {siALKBH5/siNC} | Log2Ratio {siALKBH5/siNC} | *P*-value |
| MFAP5 | 2340.3 | 291.4 | 8.0318 | 3.0057 | 0.0001 |  | 3368.7 | 291.4 | 11.5615 | 3.5313 | 0.0001 |
| PI3 | 3895.6 | 669.0 | 5.8233 | 2.5418 | 0.0000 |  | 1353.3 | 669.0 | 2.0229 | 1.0165 | 0.0009 |
| CHAC1 | 535.1 | 183.6 | 2.9141 | 1.5430 | 0.0003 |  | 872.7 | 183.6 | 4.7525 | 2.2487 | 0.0000 |
| ASNS | 1839.3 | 593.7 | 3.0982 | 1.6314 | 0.0005 |  | 2421.3 | 593.7 | 4.0786 | 2.0281 | 0.0001 |
| PYCR1 | 1145.0 | 560.5 | 2.0430 | 1.0307 | 0.0023 |  | 2054.5 | 560.5 | 3.6656 | 1.8741 | 0.0001 |
| SERPINB7 | 507.7 | 208.5 | 2.4353 | 1.2841 | 0.0027 |  | 665.7 | 208.5 | 3.1934 | 1.6751 | 0.0012 |
| THBD | 39.5 | 20.9 | 1.8846 | 0.9143 | 0.0000 |  | 77.9 | 20.9 | 3.7172 | 1.8942 | 0.0000 |
| OSBP2 | 513.0 | 144.5 | 3.5504 | 1.8280 | 0.0000 |  | 295.8 | 144.5 | 2.0474 | 1.0338 | 0.0009 |
| KRT6A | 10712.9 | 2670.7 | 4.0113 | 2.0041 | 0.0003 |  | 4192.8 | 2670.7 | 1.5699 | 0.6507 | 0.0050 |
| HMGA2 | 317.3 | 94.3 | 3.3641 | 1.7502 | 0.0001 |  | 205.1 | 94.3 | 2.1746 | 1.1208 | 0.0000 |
| E2F1 | 603.1 | 258.5 | 2.3333 | 1.2224 | 0.0008 |  | 797.6 | 258.5 | 3.0861 | 1.6258 | 0.0002 |
| QSOX2 | 211.5 | 97.0 | 2.1812 | 1.1251 | 0.0014 |  | 262.1 | 97.0 | 2.7029 | 1.4345 | 0.0006 |
| P2RX5 | 900.5 | 312.1 | 2.8857 | 1.5289 | 0.0009 |  | 615.6 | 312.1 | 1.9728 | 0.9802 | 0.0037 |
| MARCH4 | 1604.6 | 485.1 | 3.3076 | 1.7258 | 0.0012 |  | 751.8 | 485.1 | 1.5496 | 0.6319 | 0.0095 |
| FAM43A | 165.8 | 69.1 | 2.3990 | 1.2625 | 0.0041 |  | 156.2 | 69.1 | 2.2593 | 1.1759 | 0.0090 |
| CCNE1 | 1532.0 | 575.0 | 2.6645 | 1.4139 | 0.0007 |  | 1083.8 | 575.0 | 1.8850 | 0.9145 | 0.0019 |
| PVRL3 | 3839.4 | 1483.1 | 2.5887 | 1.3722 | 0.0009 |  | 2797.1 | 1483.1 | 1.8859 | 0.9153 | 0.0025 |
| SLC7A1 | 8259.7 | 4785.5 | 1.7260 | 0.7874 | 0.0077 |  | 12779.3 | 4785.5 | 2.6704 | 1.4171 | 0.0007 |
| ENDOV | 91.1 | 44.8 | 2.0307 | 1.0220 | 0.0057 |  | 105.6 | 44.8 | 2.3544 | 1.2353 | 0.0015 |
| EFNB2 | 1491.3 | 815.2 | 1.8293 | 0.8713 | 0.0066 |  | 2082.5 | 815.2 | 2.5545 | 1.3531 | 0.0005 |
| TUBB3 | 599.4 | 365.6 | 1.6393 | 0.7131 | 0.0034 |  | 989.2 | 365.6 | 2.7056 | 1.4359 | 0.0014 |
| PSAT1 | 32827.4 | 17611.5 | 1.8640 | 0.8984 | 0.0035 |  | 43022.1 | 17611.5 | 2.4428 | 1.2886 | 0.0006 |
| SLC1A5 | 4897.1 | 2542.5 | 1.9261 | 0.9457 | 0.0001 |  | 6023.3 | 2542.5 | 2.3690 | 1.2443 | 0.0001 |
| P2RX5-TAX1BP3 | 868.9 | 369.2 | 2.3533 | 1.2347 | 0.0034 |  | 704.9 | 369.2 | 1.9091 | 0.9329 | 0.0078 |
| DNMT3B | 101.4 | 45.9 | 2.2087 | 1.1432 | 0.0005 |  | 92.6 | 45.9 | 2.0187 | 1.0134 | 0.0013 |
| NGRN | 4693.2 | 2003.6 | 2.3424 | 1.2280 | 0.0001 |  | 3753.1 | 2003.6 | 1.8732 | 0.9055 | 0.0001 |
| LRRC8A | 3495.7 | 1537.9 | 2.2730 | 1.1846 | 0.0000 |  | 2876.9 | 1537.9 | 1.8707 | 0.9036 | 0.0008 |
| SPECC1L-ADORA2A | 226.2 | 115.4 | 1.9598 | 0.9707 | 0.0098 |  | 249.7 | 115.4 | 2.1634 | 1.1133 | 0.0064 |
| SCARA3 | 1313.7 | 858.0 | 1.5312 | 0.6146 | 0.0091 |  | 2223.8 | 858.0 | 2.5919 | 1.3740 | 0.0003 |
| JAG1 | 1563.4 | 670.5 | 2.3317 | 1.2214 | 0.0010 |  | 1183.1 | 670.5 | 1.7644 | 0.8192 | 0.0062 |
| FAM129A | 891.5 | 504.9 | 1.7657 | 0.8202 | 0.0065 |  | 1146.7 | 504.9 | 2.2711 | 1.1834 | 0.0018 |
| FAM118A | 807.2 | 517.3 | 1.5603 | 0.6418 | 0.0092 |  | 1264.4 | 517.3 | 2.4441 | 1.2893 | 0.0011 |
| CCND3 | 4131.1 | 2023.2 | 2.0419 | 1.0299 | 0.0001 |  | 3957.8 | 2023.2 | 1.9563 | 0.9681 | 0.0002 |
| MET | 23971.1 | 13840.1 | 1.7320 | 0.7924 | 0.0003 |  | 30738.5 | 13840.1 | 2.2210 | 1.1512 | 0.0001 |
| CENPV | 141.5 | 76.1 | 1.8594 | 0.8948 | 0.0051 |  | 154.5 | 76.1 | 2.0293 | 1.0210 | 0.0030 |
| SIRT7 | 800.2 | 428.7 | 1.8666 | 0.9004 | 0.0005 |  | 865.4 | 428.7 | 2.0188 | 1.0135 | 0.0000 |
| RAD54L2 | 251.5 | 141.2 | 1.7810 | 0.8327 | 0.0053 |  | 292.8 | 141.2 | 2.0734 | 1.0520 | 0.0043 |
| PTPMT1 | 1665.2 | 915.3 | 1.8194 | 0.8635 | 0.0081 |  | 1843.6 | 915.3 | 2.0143 | 1.0103 | 0.0028 |
| GADD45A | 3207.0 | 2109.7 | 1.5201 | 0.6042 | 0.0083 |  | 4867.5 | 2109.7 | 2.3072 | 1.2061 | 0.0007 |
| PCK2 | 182.6 | 93.2 | 1.9583 | 0.9696 | 0.0036 |  | 173.6 | 93.2 | 1.8617 | 0.8967 | 0.0076 |
| PPT2 | 2576.4 | 1611.3 | 1.5990 | 0.6771 | 0.0011 |  | 3526.1 | 1611.3 | 2.1883 | 1.1298 | 0.0004 |
| PMAIP1 | 264.4 | 157.9 | 1.6746 | 0.7438 | 0.0024 |  | 328.5 | 157.9 | 2.0807 | 1.0571 | 0.0010 |
| AKAP12 | 521.9 | 323.5 | 1.6136 | 0.6903 | 0.0019 |  | 687.4 | 323.5 | 2.1251 | 1.0875 | 0.0003 |
| NLE1 | 253.4 | 144.7 | 1.7515 | 0.8086 | 0.0089 |  | 285.4 | 144.7 | 1.9728 | 0.9802 | 0.0031 |
| SUN3 | 89.9 | 48.9 | 1.8376 | 0.8779 | 0.0057 |  | 88.7 | 48.9 | 1.8132 | 0.8585 | 0.0060 |
| INA | 24518.8 | 13219.8 | 1.8547 | 0.8912 | 0.0015 |  | 23619.6 | 13219.8 | 1.7867 | 0.8373 | 0.0000 |
| AIFM2 | 901.2 | 543.2 | 1.6589 | 0.7302 | 0.0075 |  | 1072.3 | 543.2 | 1.9739 | 0.9811 | 0.0088 |
| IFNE | 945.9 | 468.6 | 2.0186 | 1.0134 | 0.0001 |  | 752.0 | 468.6 | 1.6048 | 0.6824 | 0.0028 |
| TEKT4P2 | 932.2 | 546.1 | 1.7070 | 0.7715 | 0.0004 |  | 1024.8 | 546.1 | 1.8766 | 0.9081 | 0.0024 |
| SLC25A19 | 490.4 | 288.1 | 1.7022 | 0.7674 | 0.0007 |  | 539.6 | 288.1 | 1.8730 | 0.9053 | 0.0009 |
| MCFD2 | 13645.2 | 6698.0 | 2.0372 | 1.0266 | 0.0010 |  | 10269.6 | 6698.0 | 1.5332 | 0.6166 | 0.0015 |
| MAP1B | 28879.6 | 14890.6 | 1.9395 | 0.9557 | 0.0073 |  | 24096.9 | 14890.6 | 1.6183 | 0.6944 | 0.0078 |
| ODC1 | 10996.5 | 5756.2 | 1.9104 | 0.9338 | 0.0008 |  | 9376.9 | 5756.2 | 1.6290 | 0.7040 | 0.0048 |
| GARS | 18353.6 | 11081.3 | 1.6563 | 0.7279 | 0.0008 |  | 20740.7 | 11081.3 | 1.8717 | 0.9043 | 0.0004 |
| ZNF280B | 330.2 | 210.7 | 1.5672 | 0.6482 | 0.0023 |  | 409.9 | 210.7 | 1.9453 | 0.9600 | 0.0049 |
| MFI2 | 2016.7 | 1197.6 | 1.6839 | 0.7518 | 0.0013 |  | 2144.8 | 1197.6 | 1.7908 | 0.8406 | 0.0009 |
| DNM1L | 3152.7 | 1871.7 | 1.6845 | 0.7523 | 0.0022 |  | 3334.8 | 1871.7 | 1.7817 | 0.8333 | 0.0017 |
| TRIB3 | 1961.1 | 1083.2 | 1.8104 | 0.8563 | 0.0050 |  | 1775.1 | 1083.2 | 1.6387 | 0.7126 | 0.0030 |
| PRDM11 | 50.3 | 28.0 | 1.7950 | 0.8440 | 0.0011 |  | 46.2 | 28.0 | 1.6504 | 0.7228 | 0.0061 |
| CSF2RA | 834.2 | 484.9 | 1.7203 | 0.7826 | 0.0000 |  | 819.8 | 484.9 | 1.6906 | 0.7575 | 0.0002 |
| YARS | 4461.2 | 2890.7 | 1.5433 | 0.6260 | 0.0007 |  | 5358.4 | 2890.7 | 1.8536 | 0.8904 | 0.0003 |
| WASF3 | 55.2 | 33.1 | 1.6689 | 0.7389 | 0.0052 |  | 56.6 | 33.1 | 1.7112 | 0.7750 | 0.0030 |
| KIAA0930 | 5303.8 | 3217.5 | 1.6484 | 0.7211 | 0.0002 |  | 5446.8 | 3217.5 | 1.6928 | 0.7594 | 0.0006 |
| SPR | 2005.0 | 1192.8 | 1.6809 | 0.7492 | 0.0046 |  | 1953.4 | 1192.8 | 1.6376 | 0.7116 | 0.0020 |
| NOP16 | 1853.4 | 1179.1 | 1.5718 | 0.6524 | 0.0001 |  | 2049.7 | 1179.1 | 1.7383 | 0.7977 | 0.0000 |
| MRGBP | 103.9 | 60.3 | 1.7229 | 0.7849 | 0.0043 |  | 95.7 | 60.3 | 1.5866 | 0.6659 | 0.0041 |
| RANGRF | 410.2 | 234.4 | 1.7501 | 0.8074 | 0.0035 |  | 363.6 | 234.4 | 1.5512 | 0.6333 | 0.0060 |
| CARM1 | 5564.2 | 3395.5 | 1.6387 | 0.7125 | 0.0003 |  | 5641.3 | 3395.5 | 1.6614 | 0.7324 | 0.0005 |
| NPDC1 | 2425.0 | 1535.6 | 1.5792 | 0.6592 | 0.0015 |  | 2630.4 | 1535.6 | 1.7130 | 0.7765 | 0.0034 |
| DAP | 6045.5 | 3801.9 | 1.5901 | 0.6691 | 0.0051 |  | 6460.0 | 3801.9 | 1.6991 | 0.7648 | 0.0033 |
| HTR1D | 1342.6 | 796.7 | 1.6853 | 0.7530 | 0.0000 |  | 1275.4 | 796.7 | 1.6009 | 0.6789 | 0.0019 |
| CDKN1A | 927.0 | 603.9 | 1.5351 | 0.6183 | 0.0041 |  | 1049.8 | 603.9 | 1.7384 | 0.7977 | 0.0031 |
| WARS | 2162.8 | 1264.5 | 1.7104 | 0.7744 | 0.0012 |  | 1973.5 | 1264.5 | 1.5607 | 0.6422 | 0.0017 |
| TIMP3 | 24180.6 | 14053.0 | 1.7207 | 0.7830 | 0.0020 |  | 21530.3 | 14053.0 | 1.5321 | 0.6155 | 0.0002 |
| CLN6 | 2071.9 | 1261.2 | 1.6428 | 0.7162 | 0.0005 |  | 2029.7 | 1261.2 | 1.6093 | 0.6865 | 0.0093 |
| AARS | 10964.8 | 6746.5 | 1.6253 | 0.7007 | 0.0012 |  | 10585.8 | 6746.5 | 1.5691 | 0.6499 | 0.0012 |
| FAM219A | 256.5 | 152.7 | 1.6795 | 0.7481 | 0.0033 |  | 230.2 | 152.7 | 1.5078 | 0.5925 | 0.0069 |
| DEDD2 | 567.4 | 369.9 | 1.5341 | 0.6174 | 0.0021 |  | 606.4 | 369.9 | 1.6396 | 0.7133 | 0.0028 |
| PXK | 1063.1 | 642.1 | 1.6557 | 0.7274 | 0.0012 |  | 966.4 | 642.1 | 1.5051 | 0.5899 | 0.0023 |
| RNMTL1 | 846.5 | 533.2 | 1.5875 | 0.6668 | 0.0088 |  | 836.4 | 533.2 | 1.5686 | 0.6495 | 0.0037 |
| UAP1 | 1959.9 | 1285.9 | 1.5242 | 0.6080 | 0.0011 |  | 2076.7 | 1285.9 | 1.6150 | 0.6916 | 0.0007 |
| IFFO2 | 367.3 | 240.0 | 1.5307 | 0.6141 | 0.0050 |  | 379.6 | 240.0 | 1.5816 | 0.6614 | 0.0040 |

**Table S5. Differentially downregulated genes in expression microarray analysis**

|  | siALKBH5 #1 vs siNC (n=3) | | | | |  | siALKBH5 #3 vs siNC (n=3) | | | | |
| --- | --- | --- | --- | --- | --- | --- | --- | --- | --- | --- | --- |
| Gene Symbol | Average value of siALKBH5 | Average value of siNC | Ratio {siALKBH5/siNC} | Log2Ratio {siALKBH5/siNC} | *P*-value |  | Average value of siALKBH5 | Average value of siNC | Ratio {siALKBH5/siNC} | Log2Ratio {siALKBH5/siNC} | *P*-value |
| ALKBH5 | 107.1 | 2827.9 | 0.0379 | -4.7226 | 0.0000 |  | 40.5 | 2827.9 | 0.0143 | -6.1243 | 0.0000 |
| METTL7A | 399.0 | 1622.9 | 0.2459 | -2.0240 | 0.0002 |  | 84.8 | 1622.9 | 0.0522 | -4.2585 | 0.0002 |
| CASP14 | 20.5 | 90.7 | 0.2259 | -2.1466 | 0.0094 |  | 18.2 | 90.7 | 0.2010 | -2.3149 | 0.0062 |
| TNFSF10 | 514.1 | 1681.0 | 0.3058 | -1.7093 | 0.0006 |  | 393.2 | 1681.0 | 0.2339 | -2.0960 | 0.0003 |
| AGR2 | 113.5 | 426.8 | 0.2659 | -1.9112 | 0.0012 |  | 123.6 | 426.8 | 0.2895 | -1.7883 | 0.0014 |
| ALDH3B2 | 316.7 | 944.3 | 0.3354 | -1.5761 | 0.0002 |  | 228.5 | 944.3 | 0.2420 | -2.0469 | 0.0000 |
| LMLN | 399.5 | 1815.3 | 0.2201 | -2.1840 | 0.0000 |  | 883.8 | 1815.3 | 0.4869 | -1.0384 | 0.0001 |
| CCNG2 | 892.1 | 1878.8 | 0.4748 | -1.0746 | 0.0032 |  | 618.6 | 1878.8 | 0.3292 | -1.6028 | 0.0007 |
| GAS2L3 | 772.9 | 3386.0 | 0.2283 | -2.1312 | 0.0003 |  | 1963.0 | 3386.0 | 0.5797 | -0.7865 | 0.0002 |
| NCCRP1 | 2064.1 | 7944.8 | 0.2598 | -1.9445 | 0.0002 |  | 4515.5 | 7944.8 | 0.5684 | -0.8151 | 0.0004 |
| AMMECR1 | 360.8 | 1017.7 | 0.3545 | -1.4961 | 0.0013 |  | 511.7 | 1017.7 | 0.5028 | -0.9919 | 0.0050 |
| APAF1 | 163.9 | 306.6 | 0.5347 | -0.9032 | 0.0036 |  | 102.8 | 306.6 | 0.3354 | -1.5761 | 0.0002 |
| FAM217B | 115.7 | 326.6 | 0.3541 | -1.4976 | 0.0011 |  | 173.5 | 326.6 | 0.5313 | -0.9125 | 0.0034 |
| EYA1 | 243.0 | 545.8 | 0.4453 | -1.1672 | 0.0011 |  | 249.0 | 545.8 | 0.4563 | -1.1320 | 0.0003 |
| ALDOC | 586.4 | 929.1 | 0.6311 | -0.6640 | 0.0008 |  | 279.5 | 929.1 | 0.3008 | -1.7331 | 0.0000 |
| RNASE1 | 3723.3 | 6670.0 | 0.5582 | -0.8411 | 0.0091 |  | 2623.2 | 6670.0 | 0.3933 | -1.3464 | 0.0016 |
| SEMA4B | 401.0 | 1039.9 | 0.3856 | -1.3747 | 0.0021 |  | 594.1 | 1039.9 | 0.5713 | -0.8077 | 0.0029 |
| ST6GALNAC3 | 27.4 | 52.5 | 0.5224 | -0.9366 | 0.0041 |  | 23.8 | 52.5 | 0.4537 | -1.1403 | 0.0019 |
| AMOT | 354.7 | 782.1 | 0.4535 | -1.1407 | 0.0011 |  | 410.1 | 782.1 | 0.5244 | -0.9313 | 0.0035 |
| BZW1 | 7357.2 | 12902.4 | 0.5702 | -0.8104 | 0.0006 |  | 5468.5 | 12902.4 | 0.4238 | -1.2384 | 0.0002 |
| AMACR | 1109.5 | 1795.8 | 0.6178 | -0.6947 | 0.0016 |  | 734.4 | 1795.8 | 0.4089 | -1.2900 | 0.0003 |
| DIAPH2 | 90.5 | 204.4 | 0.4426 | -1.1758 | 0.0050 |  | 119.7 | 204.4 | 0.5857 | -0.7717 | 0.0072 |
| ANPEP | 11453.8 | 28158.2 | 0.4068 | -1.2977 | 0.0003 |  | 17856.9 | 28158.2 | 0.6342 | -0.6571 | 0.0007 |
| SLC44A4 | 1353.1 | 2886.7 | 0.4687 | -1.0931 | 0.0001 |  | 1685.0 | 2886.7 | 0.5837 | -0.7766 | 0.0013 |
| CXCL17 | 41.1 | 89.3 | 0.4605 | -1.1187 | 0.0010 |  | 53.3 | 89.3 | 0.5963 | -0.7459 | 0.0056 |
| VPS13C | 159.5 | 281.9 | 0.5659 | -0.8215 | 0.0002 |  | 144.8 | 281.9 | 0.5137 | -0.9609 | 0.0000 |
| CNOT7 | 2491.7 | 4741.3 | 0.5255 | -0.9281 | 0.0088 |  | 2670.2 | 4741.3 | 0.5632 | -0.8283 | 0.0031 |
| PECAM1 | 87.4 | 160.3 | 0.5449 | -0.8759 | 0.0025 |  | 87.7 | 160.3 | 0.5468 | -0.8708 | 0.0054 |
| GNAL | 321.7 | 587.2 | 0.5479 | -0.8680 | 0.0010 |  | 327.2 | 587.2 | 0.5572 | -0.8438 | 0.0045 |
| SLC35F5 | 772.0 | 1472.3 | 0.5244 | -0.9313 | 0.0013 |  | 885.0 | 1472.3 | 0.6011 | -0.7344 | 0.0050 |
| FAM178B | 28.3 | 45.9 | 0.6167 | -0.6973 | 0.0054 |  | 23.4 | 45.9 | 0.5096 | -0.9726 | 0.0062 |
| TMF1 | 511.9 | 1011.2 | 0.5062 | -0.9821 | 0.0000 |  | 641.1 | 1011.2 | 0.6340 | -0.6574 | 0.0009 |
| RBM47 | 1593.1 | 3110.9 | 0.5121 | -0.9655 | 0.0002 |  | 1987.2 | 3110.9 | 0.6388 | -0.6466 | 0.0009 |
| ELF3 | 299.2 | 594.2 | 0.5034 | -0.9901 | 0.0094 |  | 388.9 | 594.2 | 0.6545 | -0.6116 | 0.0087 |
| COA5 | 556.7 | 996.9 | 0.5584 | -0.8405 | 0.0004 |  | 603.5 | 996.9 | 0.6053 | -0.7242 | 0.0006 |
| SMDT1 | 576.5 | 1049.7 | 0.5492 | -0.8645 | 0.0008 |  | 650.8 | 1049.7 | 0.6200 | -0.6896 | 0.0018 |
| MAPK10 | 17.7 | 28.9 | 0.6134 | -0.7052 | 0.0066 |  | 16.1 | 28.9 | 0.5572 | -0.8438 | 0.0066 |
| SKAP2 | 1527.6 | 2783.4 | 0.5488 | -0.8656 | 0.0004 |  | 1761.5 | 2783.4 | 0.6328 | -0.6601 | 0.0025 |
| HERC6 | 177.5 | 298.3 | 0.5952 | -0.7485 | 0.0006 |  | 186.9 | 298.3 | 0.6267 | -0.6742 | 0.0049 |
| LNPEP | 1586.6 | 2625.7 | 0.6043 | -0.7268 | 0.0034 |  | 1641.5 | 2625.7 | 0.6252 | -0.6777 | 0.0028 |
| SERINC4 | 36.3 | 55.5 | 0.6543 | -0.6120 | 0.0039 |  | 32.2 | 55.5 | 0.5795 | -0.7872 | 0.0022 |
| MYLIP | 1133.8 | 1890.9 | 0.5996 | -0.7380 | 0.0018 |  | 1201.9 | 1890.9 | 0.6356 | -0.6537 | 0.0020 |
| BNIP3L | 11304.4 | 18855.9 | 0.5995 | -0.7381 | 0.0013 |  | 12232.8 | 18855.9 | 0.6487 | -0.6243 | 0.0005 |
| FRK | 1159.5 | 1926.4 | 0.6019 | -0.7323 | 0.0037 |  | 1263.8 | 1926.4 | 0.6561 | -0.6081 | 0.0025 |
| C7orf69 | 13.1 | 20.0 | 0.6573 | -0.6053 | 0.0051 |  | 12.0 | 20.0 | 0.6021 | -0.7320 | 0.0024 |
| CAMK2A | 39.0 | 59.1 | 0.6600 | -0.5994 | 0.0072 |  | 36.1 | 59.1 | 0.6113 | -0.7101 | 0.0044 |
| TCAIM | 2174.4 | 3305.3 | 0.6578 | -0.6042 | 0.0014 |  | 2115.3 | 3305.3 | 0.6400 | -0.6439 | 0.0031 |

**Table S6. Expression of genes associated with m^6^A modification enzymes in expression microarray analysis**

|  | siALKBH5 #1 vs siNC (n=3) | | | | |  | siALKBH5 #3 vs siNC (n=3) | | | | |
| --- | --- | --- | --- | --- | --- | --- | --- | --- | --- | --- | --- |
| Demethylase | Average value of siALKBH5 | Average value of siNC | Ratio {siALKBH5/siNC} | Log2Ratio {siALKBH5/siNC} | *P*-value |  | Average value of siALKBH5 | Average value of siNC | Ratio {siALKBH5/siNC} | Log2Ratio {siALKBH5/siNC} | *P*-value |
| ALKBH5 | 107.1 | 2827.9 | 0.0379 | -4.723 | 0 |  | 40.5 | 2827.9 | 0.0143 | -6.124 | 0 |
| FTO | 5745.0 | 7136.2 | 0.8051 | -0.313 | 0.0222 |  | 5529.0 | 7136.2 | 0.7748 | -0.368 | 0.0401 |
| ALKBH3 | 494.0 | 475.3 | 1.0393 | 0.0556 | 0.6055 |  | 365.5 | 475.3 | 0.7691 | -0.379 | 0.079 |
|  |  |  |  |  |  |  |  |  |  |  |  |
| Methyltransferase complex | | |  |  |  |  |  |  |  |  |  |
| METTL3 | 961.7 | 1080.7 | 0.8899 | -0.168 | 0.3722 |  | 998.4 | 1080.7 | 0.9238 | -0.114 | 0.392 |
| METTL14 | 457.0 | 582.3 | 0.7847 | -0.35 | 0.2927 |  | 505.9 | 582.3 | 0.8688 | -0.203 | 0.5005 |
| WTAP | 1771.4 | 1564.9 | 1.1319 | 0.1788 | 0.1376 |  | 1961.1 | 1564.9 | 1.2531 | 0.3255 | 0.0582 |
| METTL5 | 1845.1 | 1374.5 | 1.3424 | 0.4248 | 0.0326 |  | 1788.4 | 1374.5 | 1.3011 | 0.3797 | 0.038 |
| METTL16 | 15.3 | 16.6 | 0.9233 | -0.115 | 0.6276 |  | 15.0 | 16.6 | 0.9047 | -0.144 | 0.5641 |
| METTL16 | 727.6 | 617.3 | 1.1787 | 0.2372 | 0.2842 |  | 964.6 | 617.3 | 1.5626 | 0.644 | 0.0662 |
| RBM15 | 1808.5 | 1637.1 | 1.1047 | 0.1437 | 0.0419 |  | 2269.2 | 1637.1 | 1.3861 | 0.4711 | 0.0217 |
| RBM15B | 506.1 | 608.5 | 0.8318 | -0.266 | 0.2391 |  | 723.6 | 608.5 | 1.1893 | 0.2501 | 0.1926 |
| CBLL1 | 8701.2 | 8906.3 | 0.977 | -0.034 | 0.709 |  | 9344.4 | 8906.3 | 1.0492 | 0.0693 | 0.6507 |
| ZC3H13 | 54.3 | 58.1 | 0.9343 | -0.098 | 0.6778 |  | 54.5 | 58.1 | 0.9374 | -0.093 | 0.7122 |
| ZC3H13 | 6184.9 | 7257.1 | 0.8523 | -0.231 | 0.095 |  | 7531.8 | 7257.1 | 1.0379 | 0.0536 | 0.5715 |
| KIAA1429 | 461.1 | 961.4 | 0.4796 | -1.06 | 0.0053 |  | 1031.1 | 961.4 | 1.0725 | 0.101 | 0.4399 |
| ZCCHC4 | 216.4 | 219.0 | 0.9883 | -0.017 | 0.8342 |  | 210.4 | 219.0 | 0.961 | -0.057 | 0.6658 |
|  |  |  |  |  |  |  |  |  |  |  |  |
| Reader |  |  |  |  |  |  |  |  |  |  |  |
| YTHDF1 | 4979.4 | 4101.1 | 1.2142 | 0.28 | 0.0446 |  | 5440.9 | 4101.1 | 1.3267 | 0.4078 | 0.0221 |
| YTHDF2 | 2433.1 | 3075.8 | 0.791 | -0.338 | 0.0208 |  | 3636.4 | 3075.8 | 1.1822 | 0.2415 | 0.0537 |
| YTHDF3 | 13190.2 | 13826.8 | 0.954 | -0.068 | 0.6715 |  | 16690.8 | 13826.8 | 1.2071 | 0.2716 | 0.1952 |
| YTHDC1 | 1308.9 | 1221.5 | 1.0716 | 0.0997 | 0.5655 |  | 1754.1 | 1221.5 | 1.436 | 0.522 | 0.0549 |
| YTHDC2 | 430.0 | 383.0 | 1.1226 | 0.1669 | 0.3652 |  | 466.1 | 383.0 | 1.217 | 0.2833 | 0.0929 |
| HNRNPA2B1 | 39293.2 | 40462.4 | 0.9711 | -0.042 | 0.6156 |  | 45872.4 | 40462.4 | 1.1337 | 0.181 | 0.0864 |
| HNRNPC | 48511.0 | 55197.7 | 0.8789 | -0.186 | 0.0067 |  | 42765.5 | 55197.7 | 0.7748 | -0.368 | 0.0007 |
| IGF2BP1 | 3615.5 | 3329.2 | 1.086 | 0.119 | 0.2764 |  | 3720.2 | 3329.2 | 1.1175 | 0.1602 | 0.175 |
| IGF2BP2 | 11701.2 | 10073.8 | 1.1615 | 0.216 | 0.1165 |  | 13712.1 | 10073.8 | 1.3612 | 0.4448 | 0.0261 |
| IGF2BP3 | 5008.1 | 4932.4 | 1.0153 | 0.022 | 0.7458 |  | 5341.9 | 4932.4 | 1.083 | 0.115 | 0.3002 |
| FMR1 | 3459.1 | 5920.5 | 0.5843 | -0.775 | 0.0003 |  | 6670.0 | 5920.5 | 1.1266 | 0.172 | 0.2414 |

**Table S7. Hypermethylated genes in epitranscriptomic microarray analysis**

|  | siALKBH5#1 vs siNC (n=3) | |  | siALKBH5#3 vs siNC (n=3) | |
| --- | --- | --- | --- | --- | --- |
| gene name | Log2-ratio {siALKBH5/siNC} | *P*-value |  | Log2-ratio {siALKBH5/siNC} | *P*-value |
| GPX6 | 1.097 | 0.02899 |  | 1.088 | 0.01105 |
| MFSD1 | 0.970 | 0.01481 |  | 0.866 | 0.03850 |
| CTD-2170G1.2 | 0.889 | 0.01686 |  | 0.879 | 0.00287 |
| MFAP5 | 0.756 | 0.01284 |  | 0.857 | 0.00322 |
| pri-3-hsa-mir-378a | 0.777 | 0.03654 |  | 0.782 | 0.03859 |
| RP11-112J3.15 | 0.703 | 0.09860 |  | 0.843 | 0.00489 |
| HPS1 | 0.700 | 0.03593 |  | 0.810 | 0.00871 |
| DGCR11 | 0.613 | 0.01739 |  | 0.834 | 0.00013 |
| MAEA | 0.664 | 0.01726 |  | 0.637 | 0.01471 |
| CREB3L4 | 0.635 | 0.02386 |  | 0.645 | 0.01583 |
| ADCY5 | 0.589 | 0.01639 |  | 0.613 | 0.00788 |
