# Supplementary figures and images for: m6A demethylase ALKBH5 promotes tumor cell proliferation by destabilizing IGF2BPs target genes and worsens the prognosis of patients with non-small-cell lung cancer
Source: Cancer Gene Ther. 2022 Mar 22;29(10):1355–72. doi: 10.1038/s41417-022-00451-8 (PMC9576599; doi:10.1038/s41417-022-00451-8)

## Slide 1
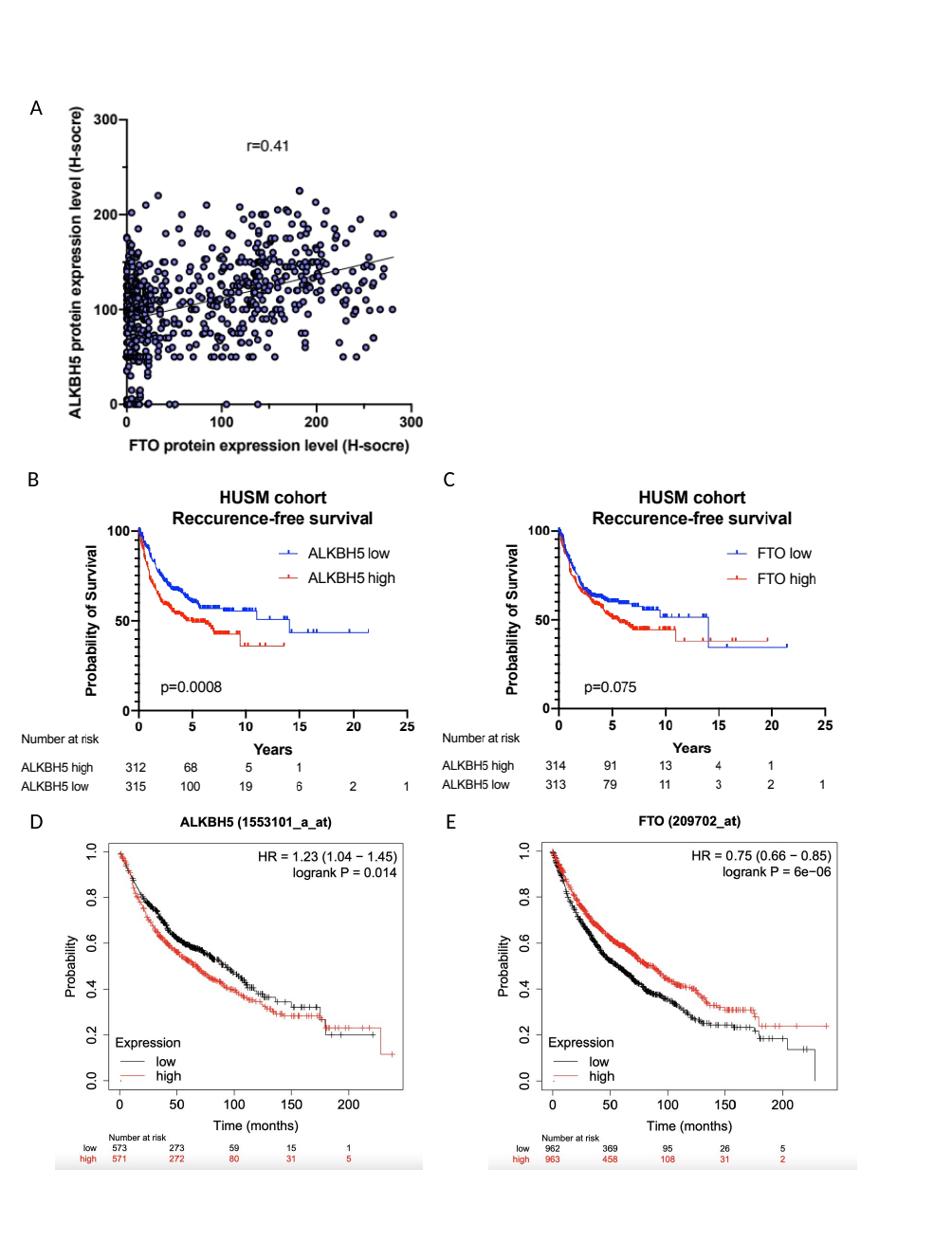

A
B
C
D
E

Supplement: Supplementary file 3 — Figure S1 [file 41417_2022_451_MOESM3_ESM.pptx]

## Slide 1
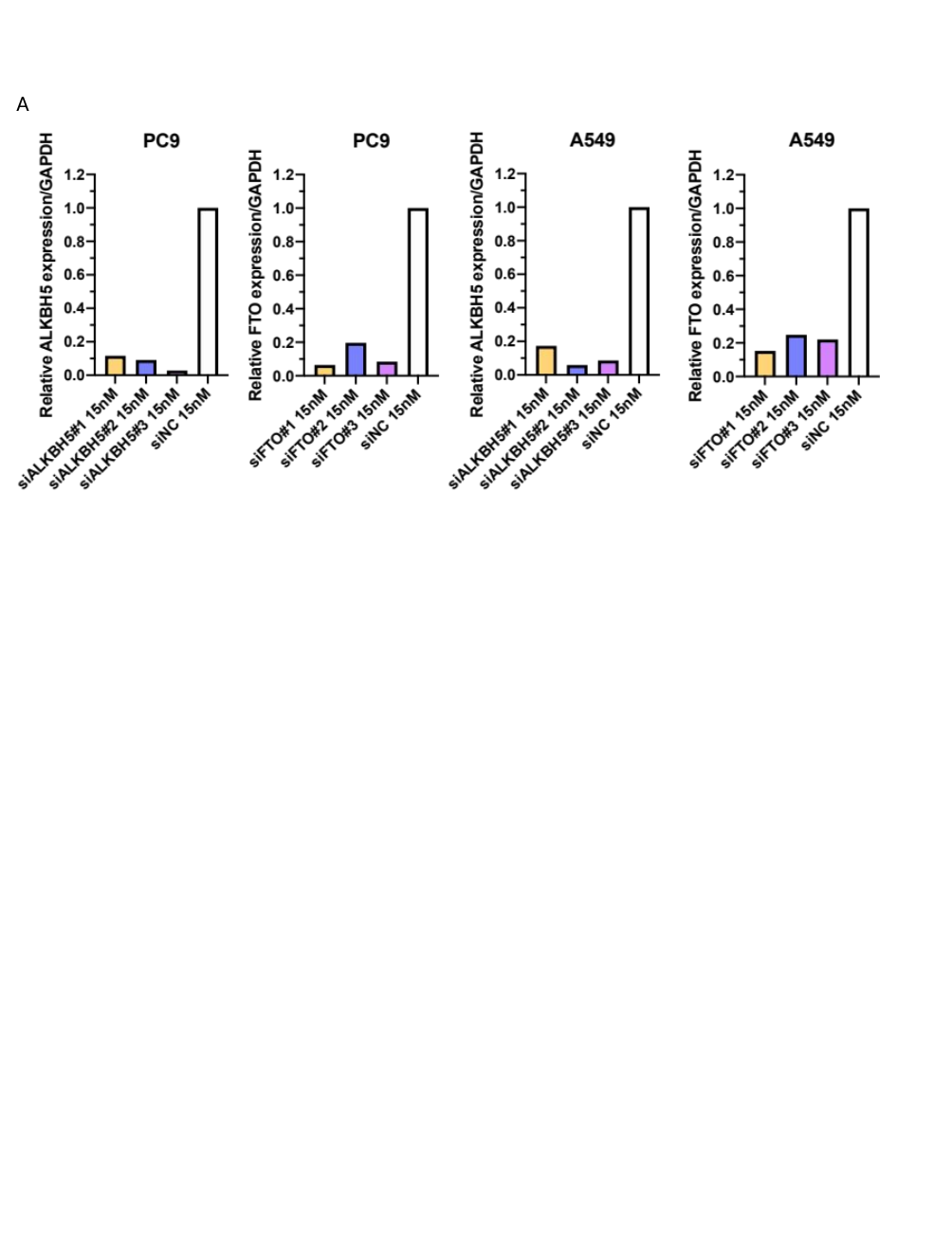

A

Supplement: Supplementary file 4 — Figure S2 [file 41417_2022_451_MOESM4_ESM.pptx]

## Slide 1
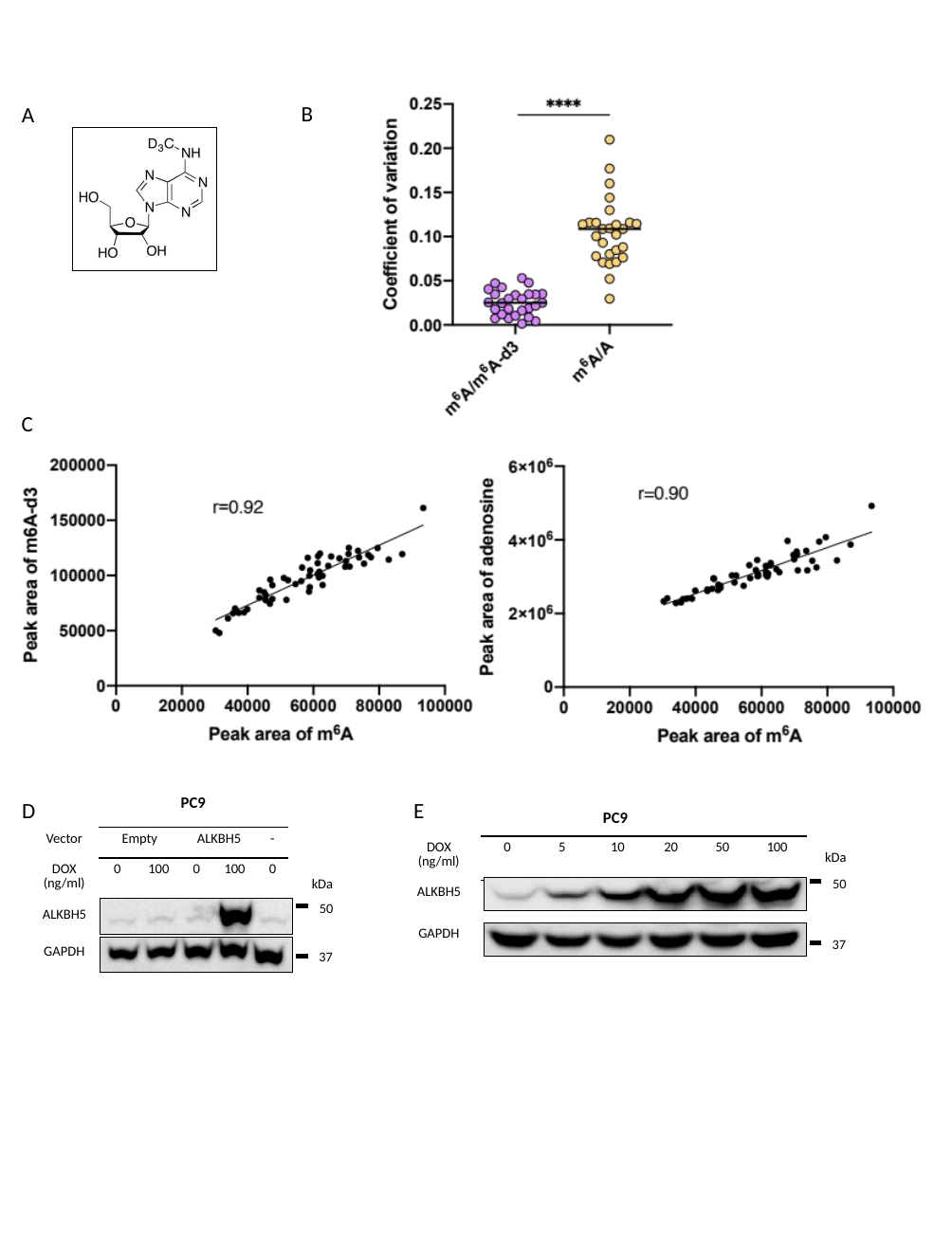

B
A
C
D
E
| | PC9 | PC9 | | | |
| --- | --- | --- | --- | --- | --- |
| Vector | Empty | | ALKBH5 | | - |
| DOX (ng/ml) | 0 | 100 | 0 | 100 | 0 |
| ALKBH5 | | | | | |
| GAPDH | | | | | |
| | | PC9 | PC9 | | | |
| --- | --- | --- | --- | --- | --- | --- |
| DOX (ng/ml) | 0 | 5 | 10 | 20 | 50 | 100 |
| ALKBH5 | | | | | | |
| GAPDH | | | | | | |
kDa
kDa
50
50
37
37

Supplement: Supplementary file 5 — Figure S3 [file 41417_2022_451_MOESM5_ESM.pptx]

## Slide 1
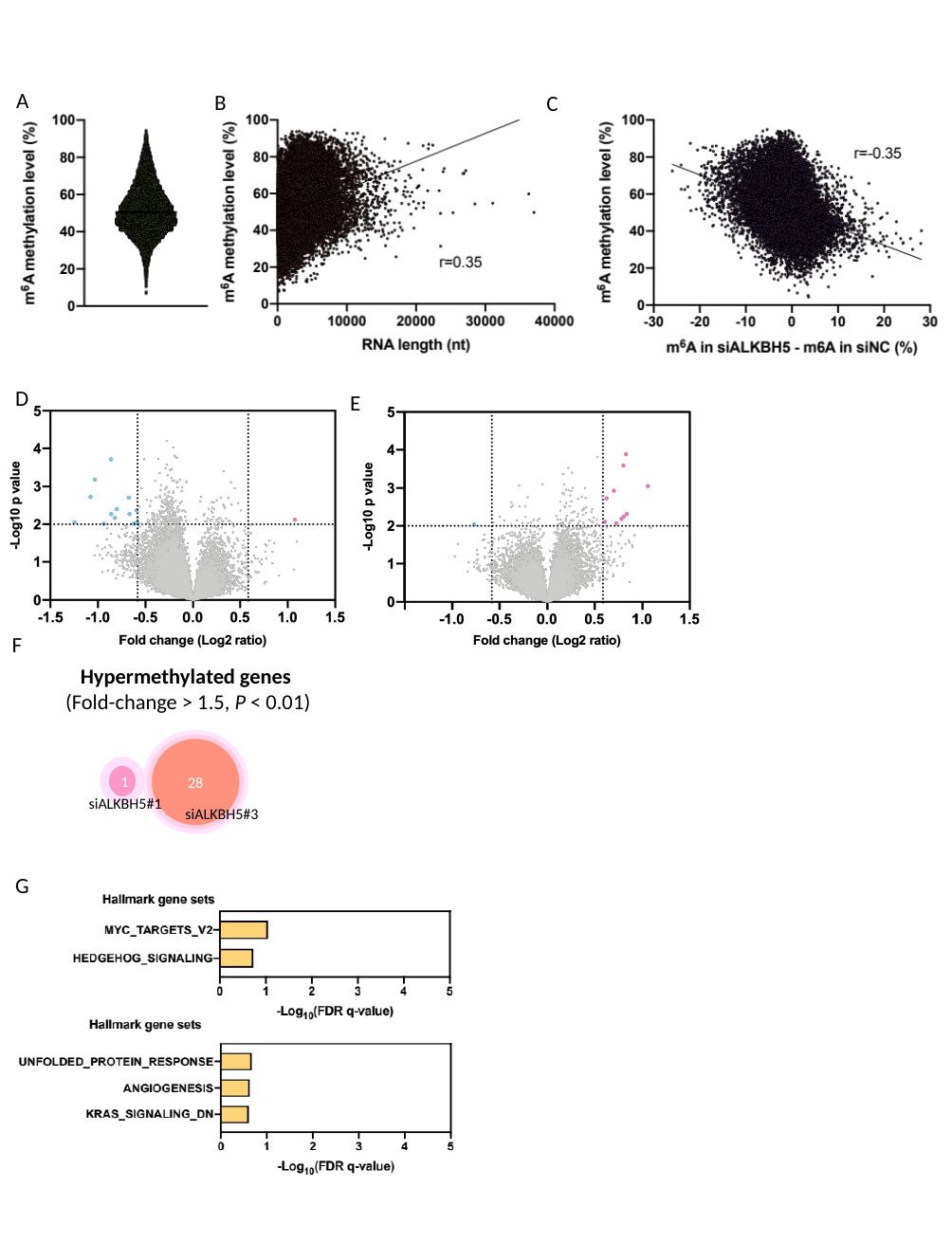

A
B
C
D
E
F
Hypermethylated genes
(Fold-change > 1.5, P < 0.01)
28
1
siALKBH5#1
siALKBH5#3
G

Supplement: Supplementary file 7 — Figure S5 [file 41417_2022_451_MOESM7_ESM.pptx]

## Slide 1
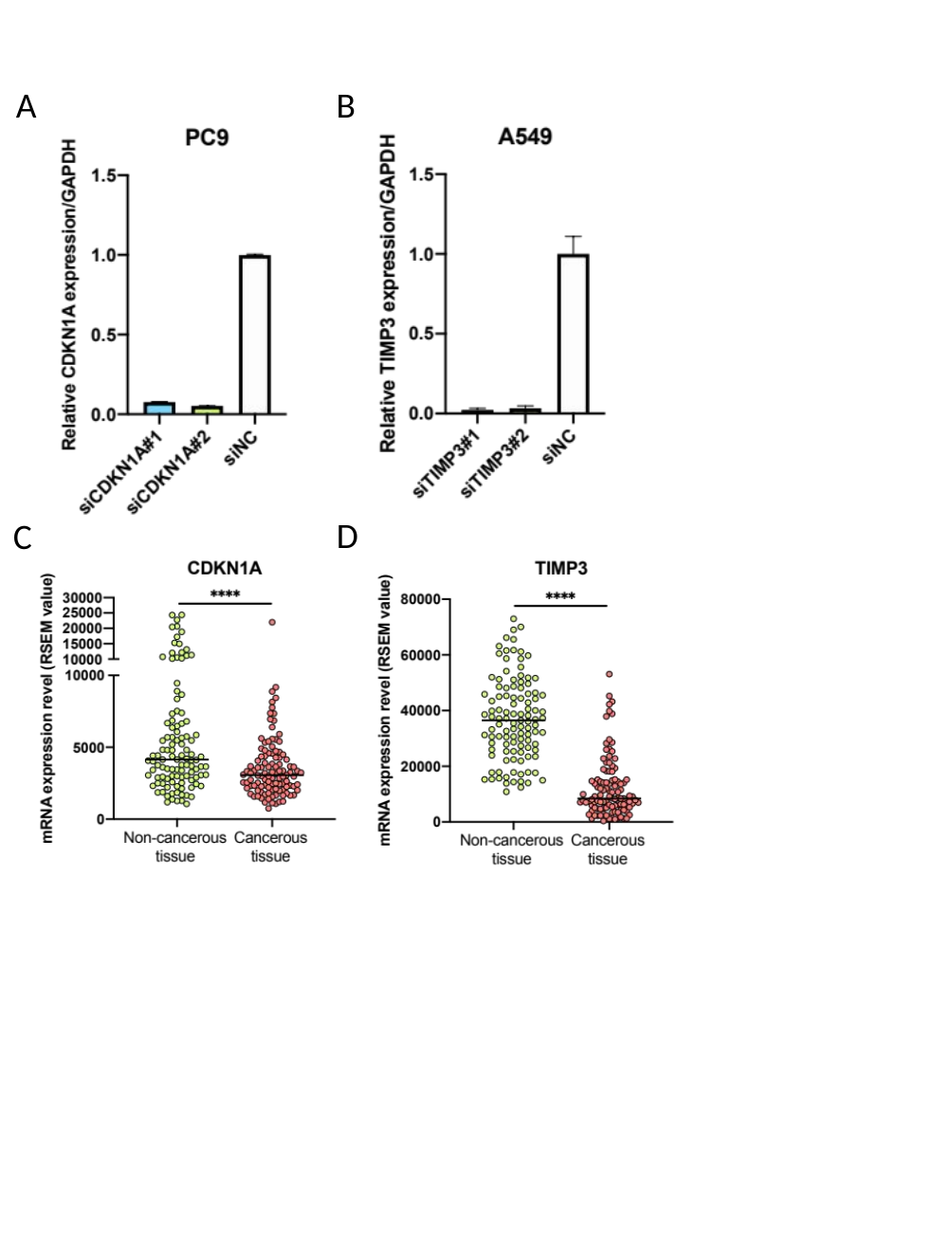

A
B
D
C

Supplement: Supplementary file 9 — Figure S7 [file 41417_2022_451_MOESM9_ESM.pptx]

## Slide 1
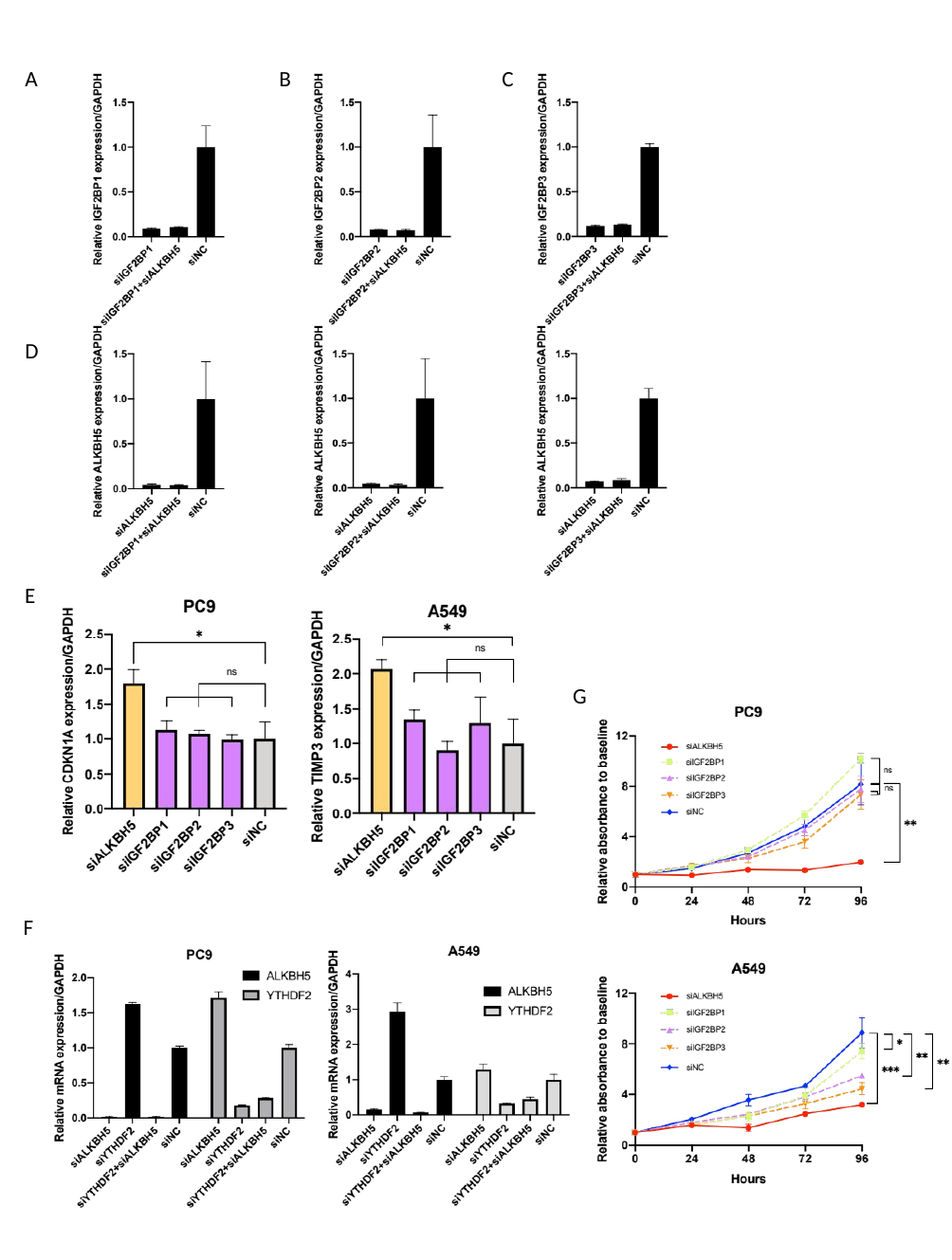

A
B
C
D
E
G
F

Supplement: Supplementary file 10 — Figure S8 [file 41417_2022_451_MOESM10_ESM.pptx]
