# Supplementary material for: m6A demethylase ALKBH5 promotes tumor cell proliferation by destabilizing IGF2BPs target genes and worsens the prognosis of patients with non-small-cell lung cancer
Source: Cancer Gene Ther. 2022 Mar 22;29(10):1355–72. doi: 10.1038/s41417-022-00451-8 (PMC9576599; doi:10.1038/s41417-022-00451-8)
Supplement: Supplementary file 6 — Figure S4 [file 41417_2022_451_MOESM6_ESM.pptx]

## Slide 1
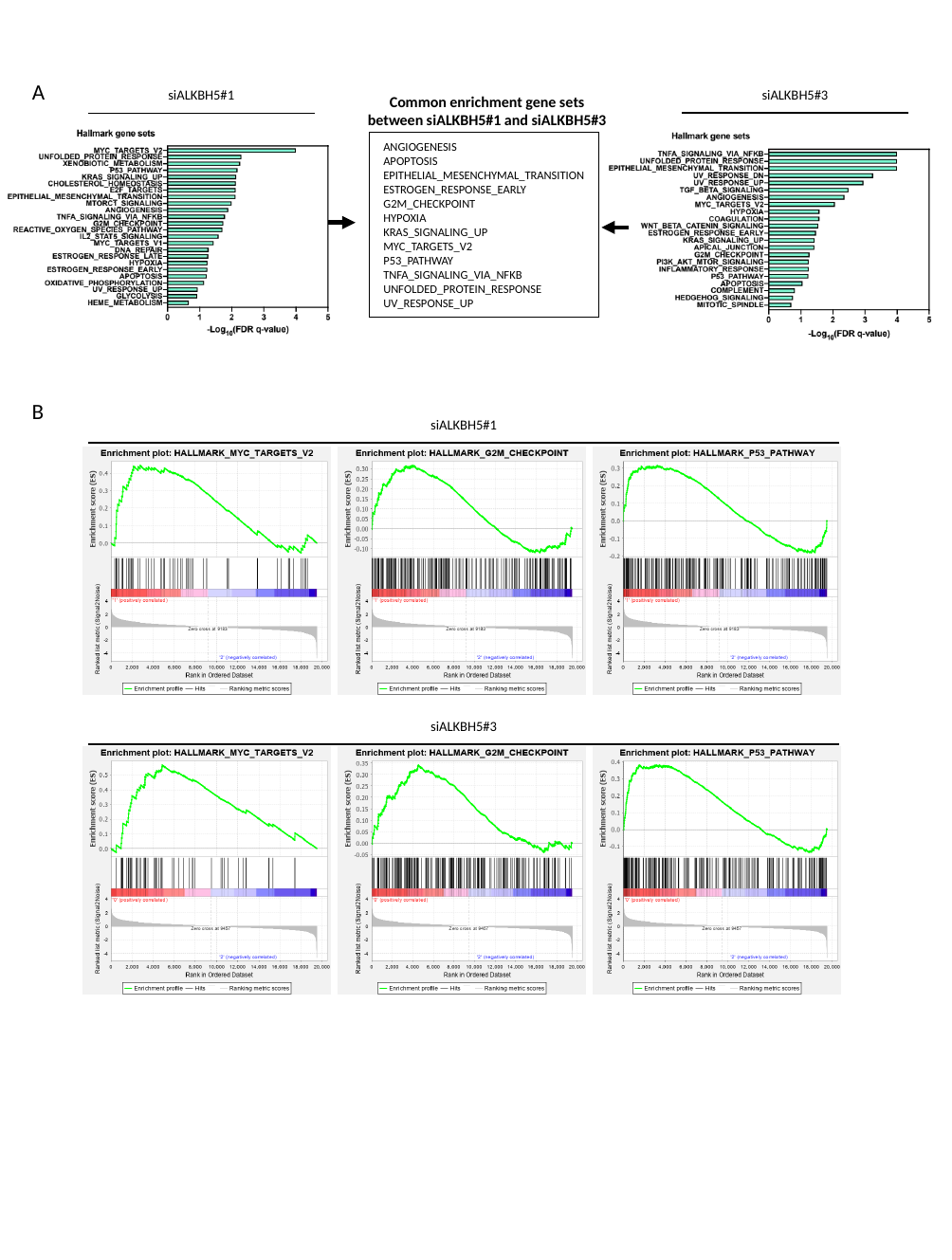

A
| siALKBH5#3 |
| --- |
| siALKBH5#1 |
| --- |
Common enrichment gene sets between siALKBH5#1 and siALKBH5#3
ANGIOGENESIS
APOPTOSIS
EPITHELIAL_MESENCHYMAL_TRANSITION
ESTROGEN_RESPONSE_EARLY
G2M_CHECKPOINT
HYPOXIA
KRAS_SIGNALING_UP
MYC_TARGETS_V2
P53_PATHWAY
TNFA_SIGNALING_VIA_NFKB
UNFOLDED_PROTEIN_RESPONSE
UV_RESPONSE_UP
B
| siALKBH5#1 |
| --- |
| siALKBH5#3 |
| --- |
