# Supplementary material for: m6A demethylase ALKBH5 promotes tumor cell proliferation by destabilizing IGF2BPs target genes and worsens the prognosis of patients with non-small-cell lung cancer
Source: Cancer Gene Ther. 2022 Mar 22;29(10):1355–72. doi: 10.1038/s41417-022-00451-8 (PMC9576599; doi:10.1038/s41417-022-00451-8)
Supplement: Supplementary file 8 — Figure S6 [file 41417_2022_451_MOESM8_ESM.pptx]

## Slide 1
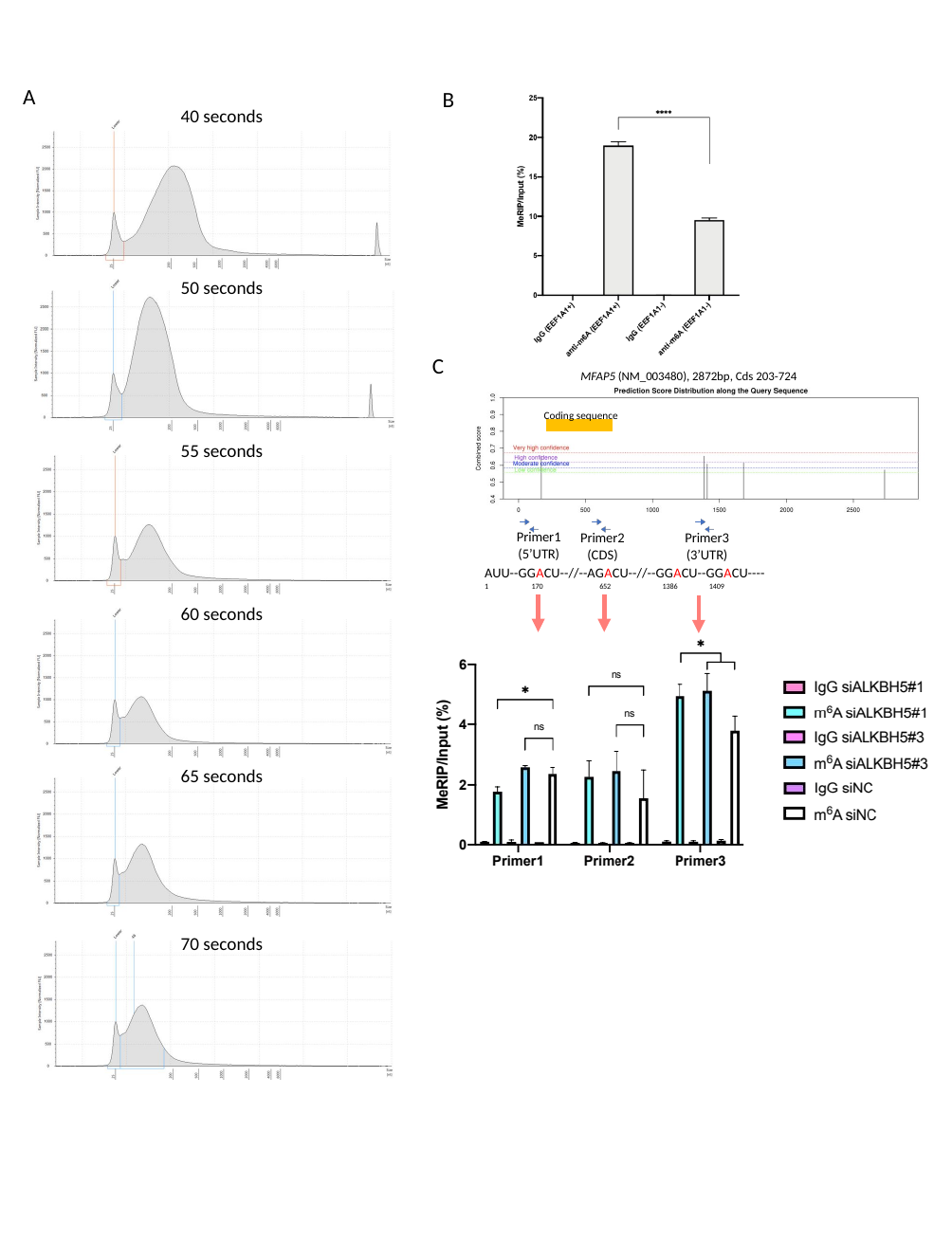

A
B
40 seconds
50 seconds
C
MFAP5 (NM_003480), 2872bp, Cds 203-724
Coding sequence
55 seconds
Primer1
(5’UTR)
Primer3
(3’UTR)
Primer2
(CDS)
AUU--GGACU--//--AGACU--//--GGACU--GGACU----
1 170 652 1386 1409
60 seconds
65 seconds
70 seconds
