# Supplementary material for: m6A demethylase ALKBH5 promotes tumor cell proliferation by destabilizing IGF2BPs target genes and worsens the prognosis of patients with non-small-cell lung cancer
Source: Cancer Gene Ther. 2022 Mar 22;29(10):1355–72. doi: 10.1038/s41417-022-00451-8 (PMC9576599; doi:10.1038/s41417-022-00451-8)

Figure 1D

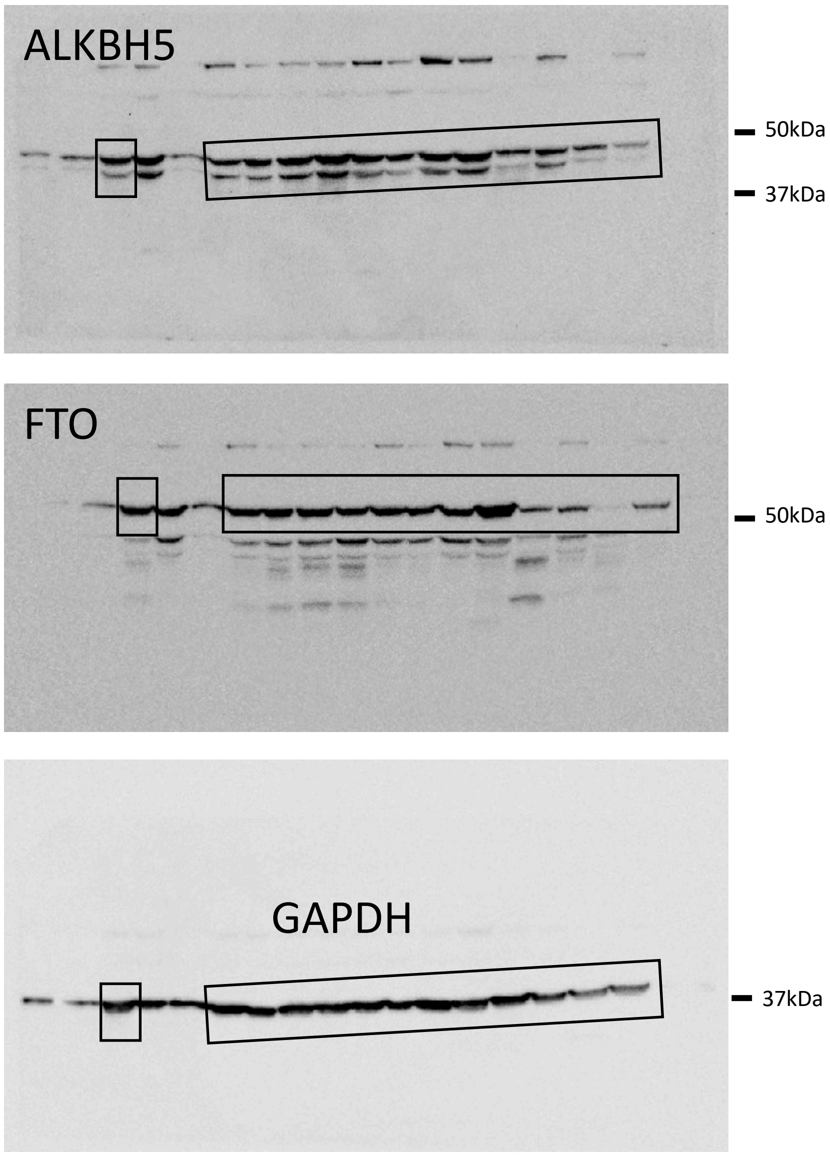

Figure 2A

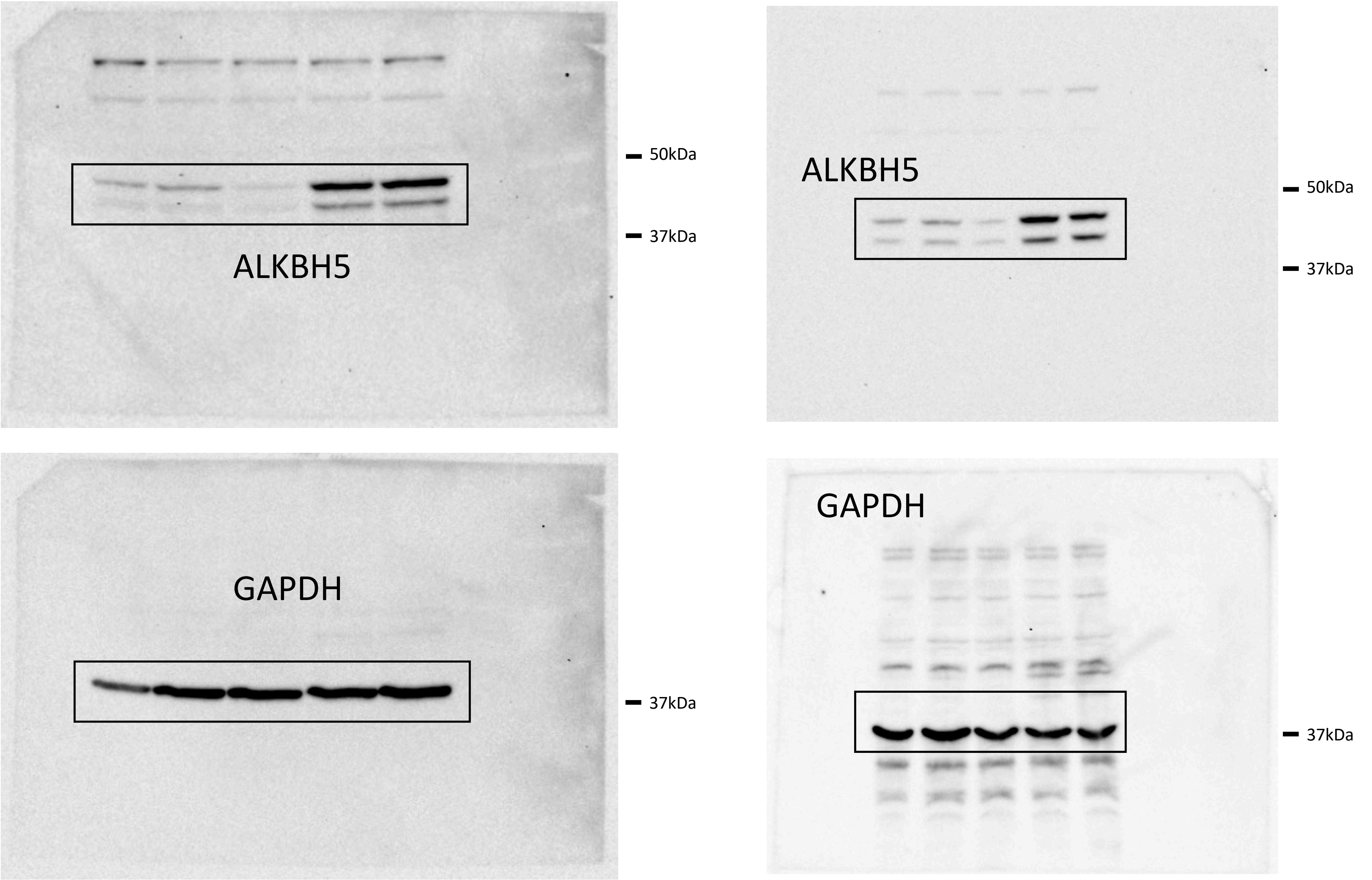
ALKBH5
50kDa
37kDa
ALKBH5
50kDa
37kDa
GAPDH
37kDa
GAPDH
37kDa

Figure 2A

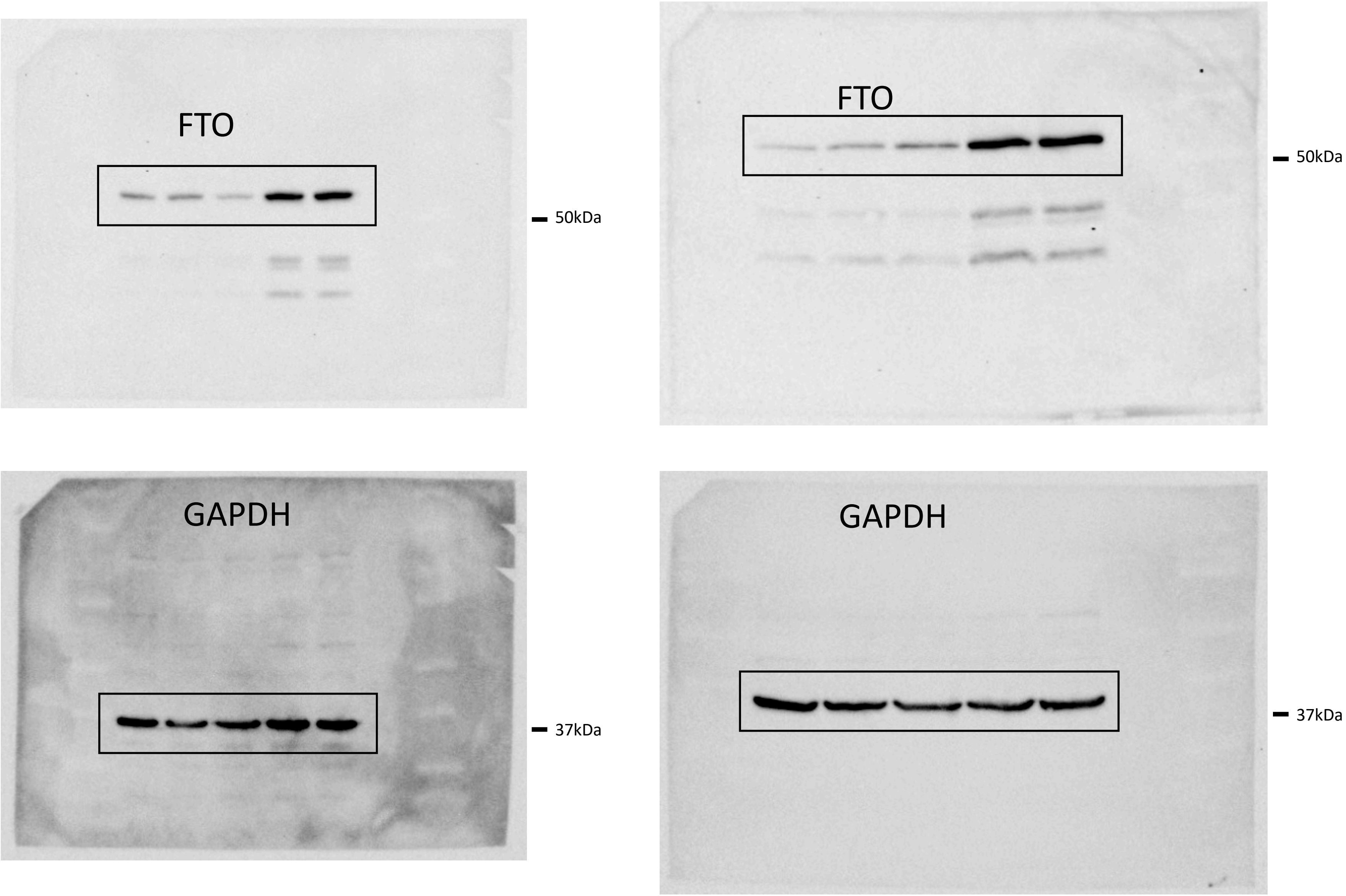

Figure 4A

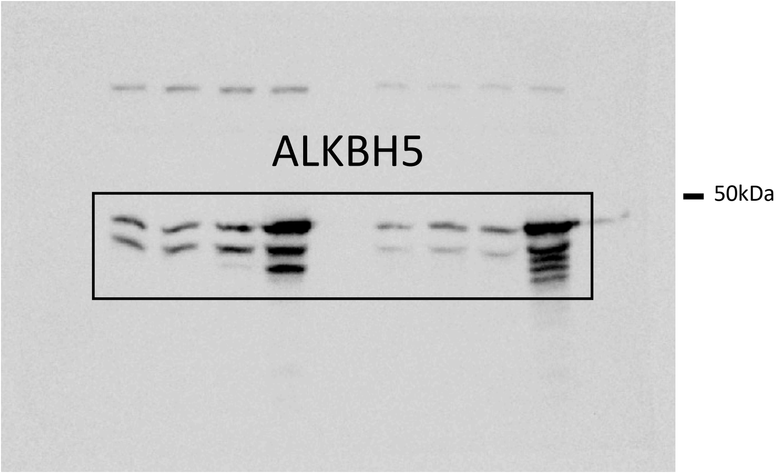

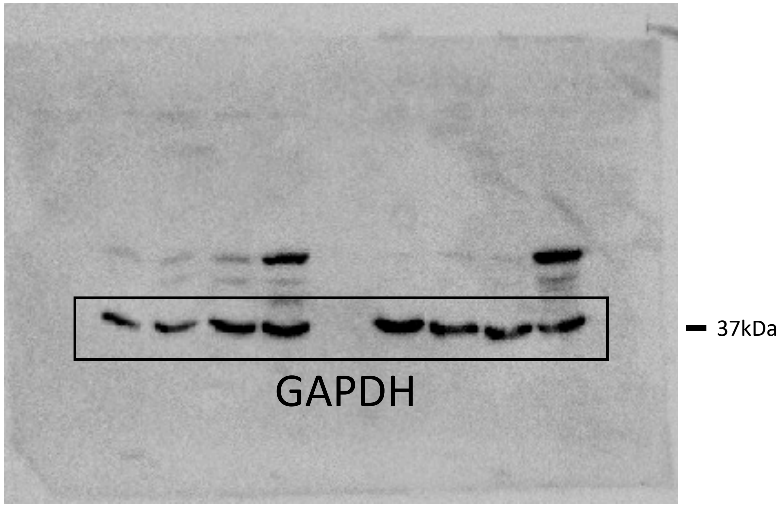

Figure 7B

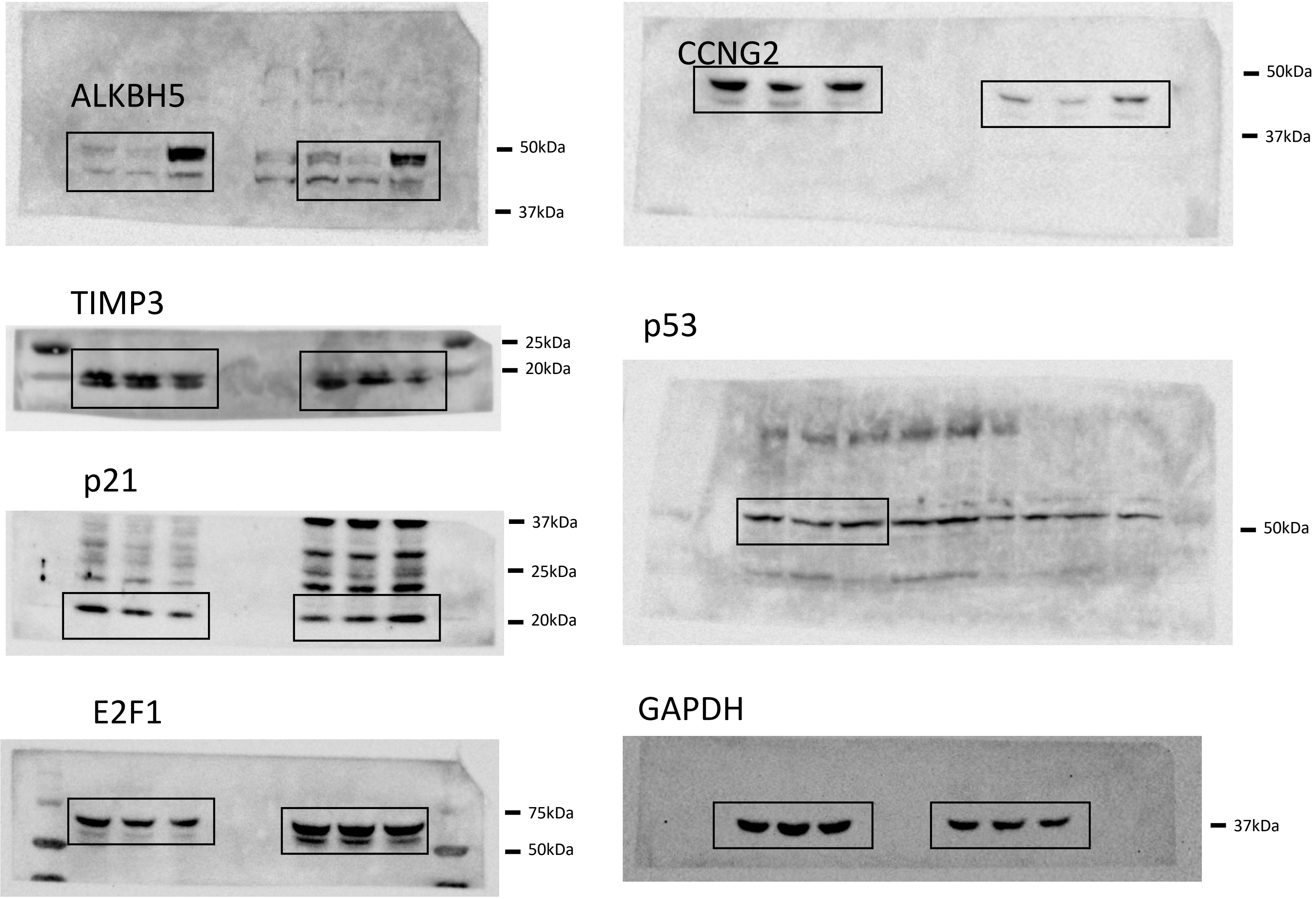
ALKBH5
50kDa
37kDa
CCNG2
50kDa
37kDa
TIMP3
25kDa
20kDa
p53
50kDa
p21
37kDa
25kDa
20kDa
E2F1
75kDa
50kDa
GAPDH
37kDa

Figure 7C and Figure 7D

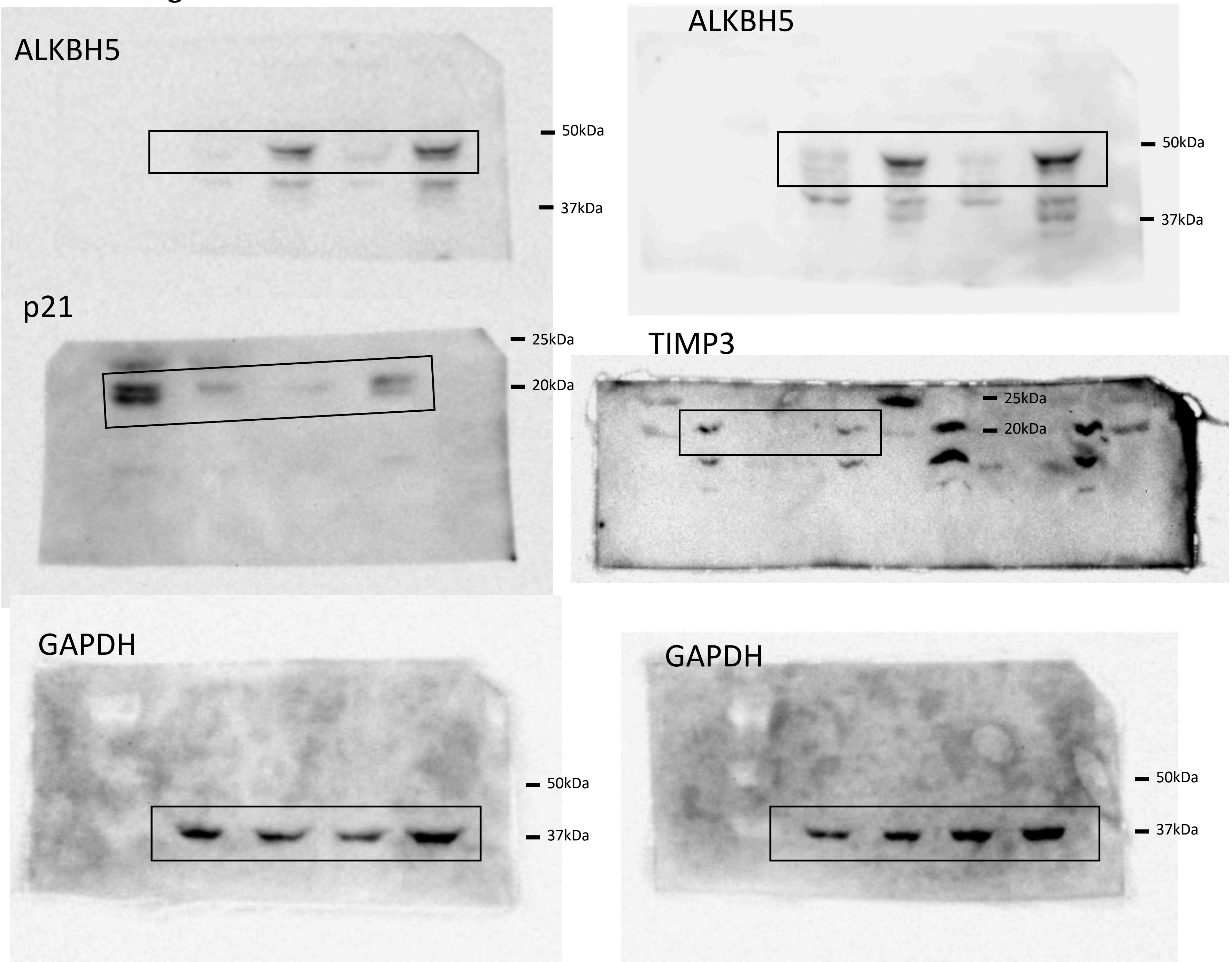

Figure 8A

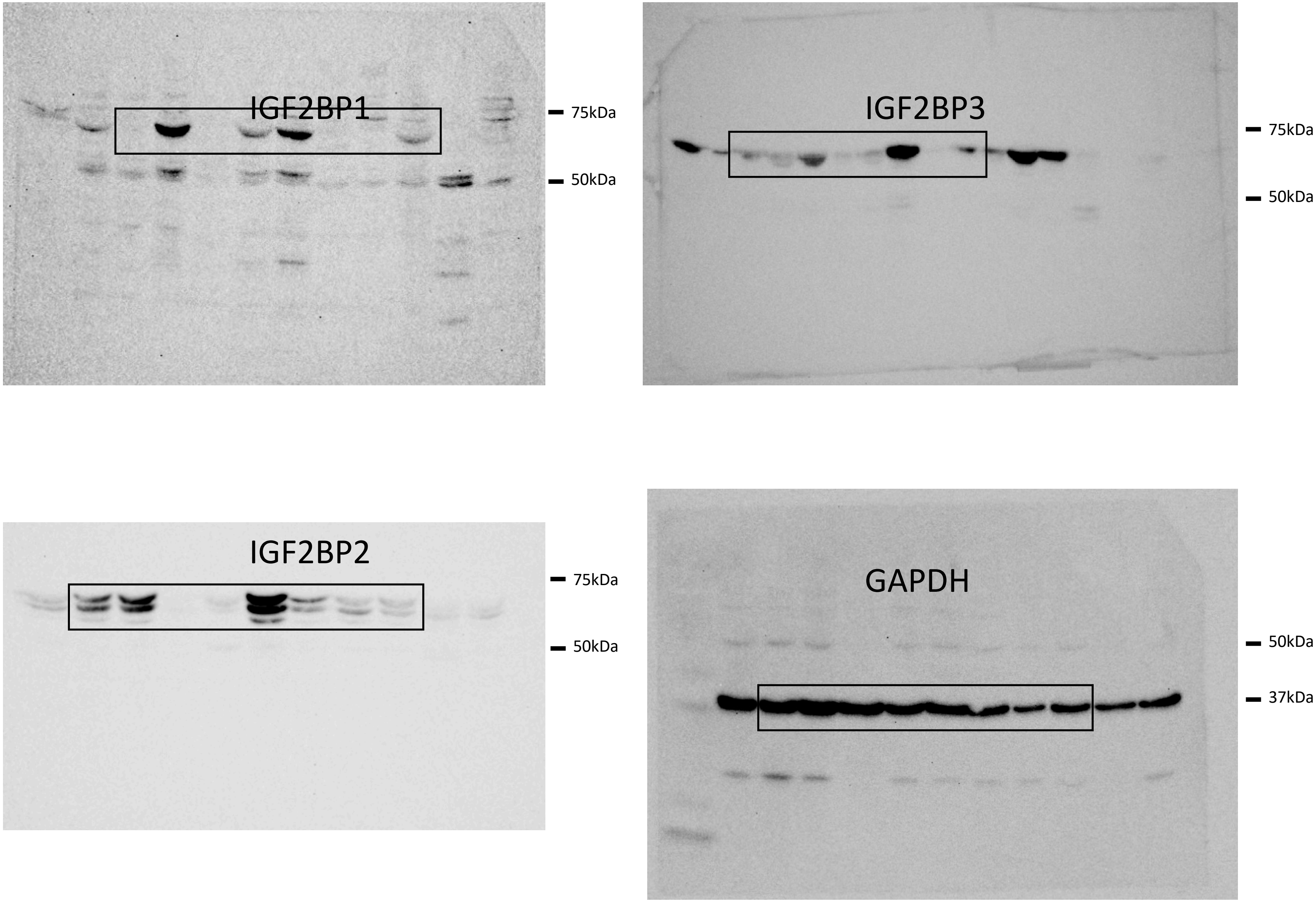
IGF2BP1
75kDa
50kDa
IGF2BP3
75kDa
50kDa
IGF2BP2
75kDa
50kDa
GAPDH
50kDa
37kDa

Figure 8B

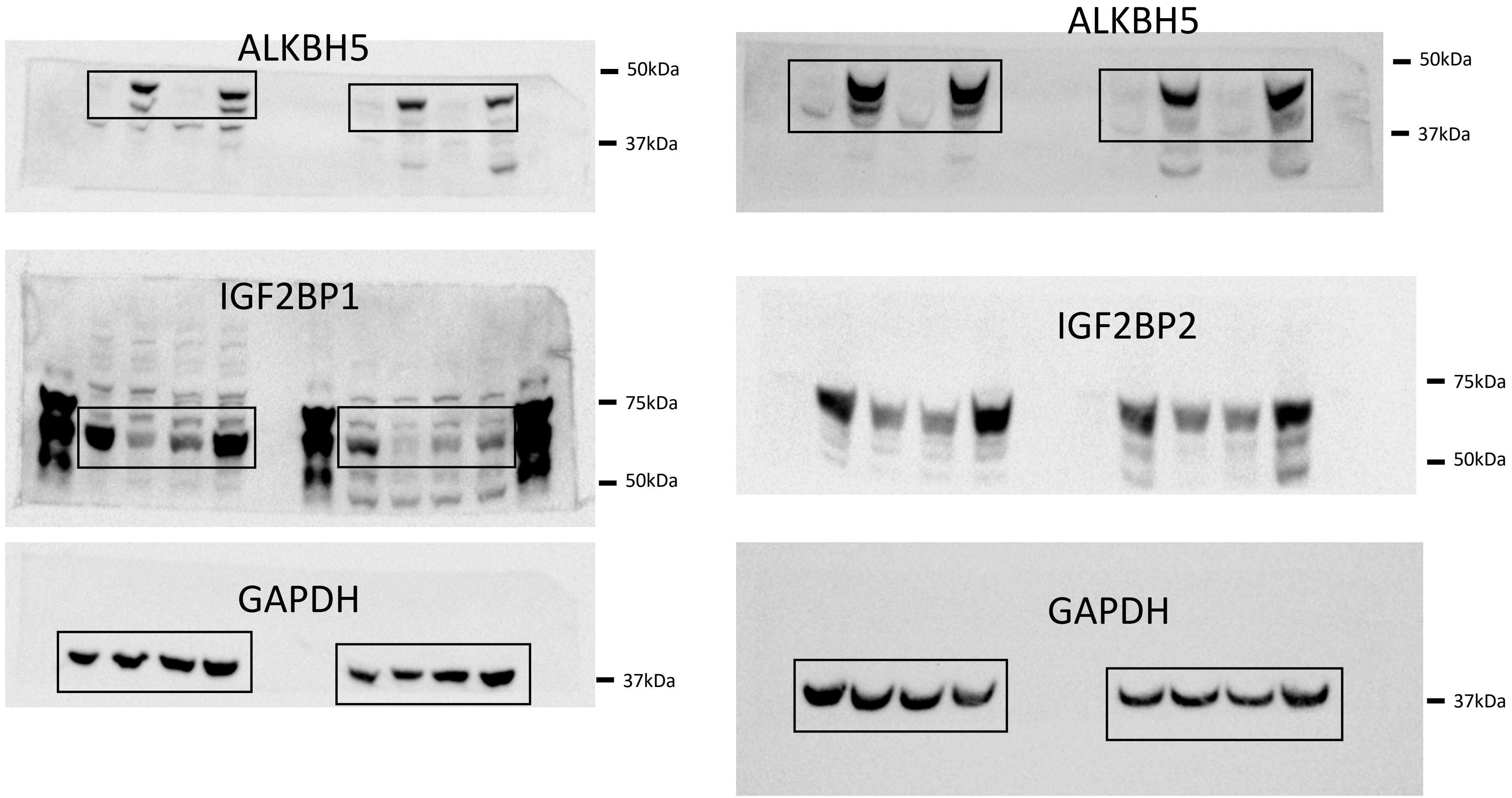
ALKBH5
50kDa
37kDa
ALKBH5
50kDa
37kDa
IGF2BP1
75kDa
50kDa
IGF2BP2
75kDa
50kDa
GAPDH
37kDa
GAPDH
37kDa

Figure 8B

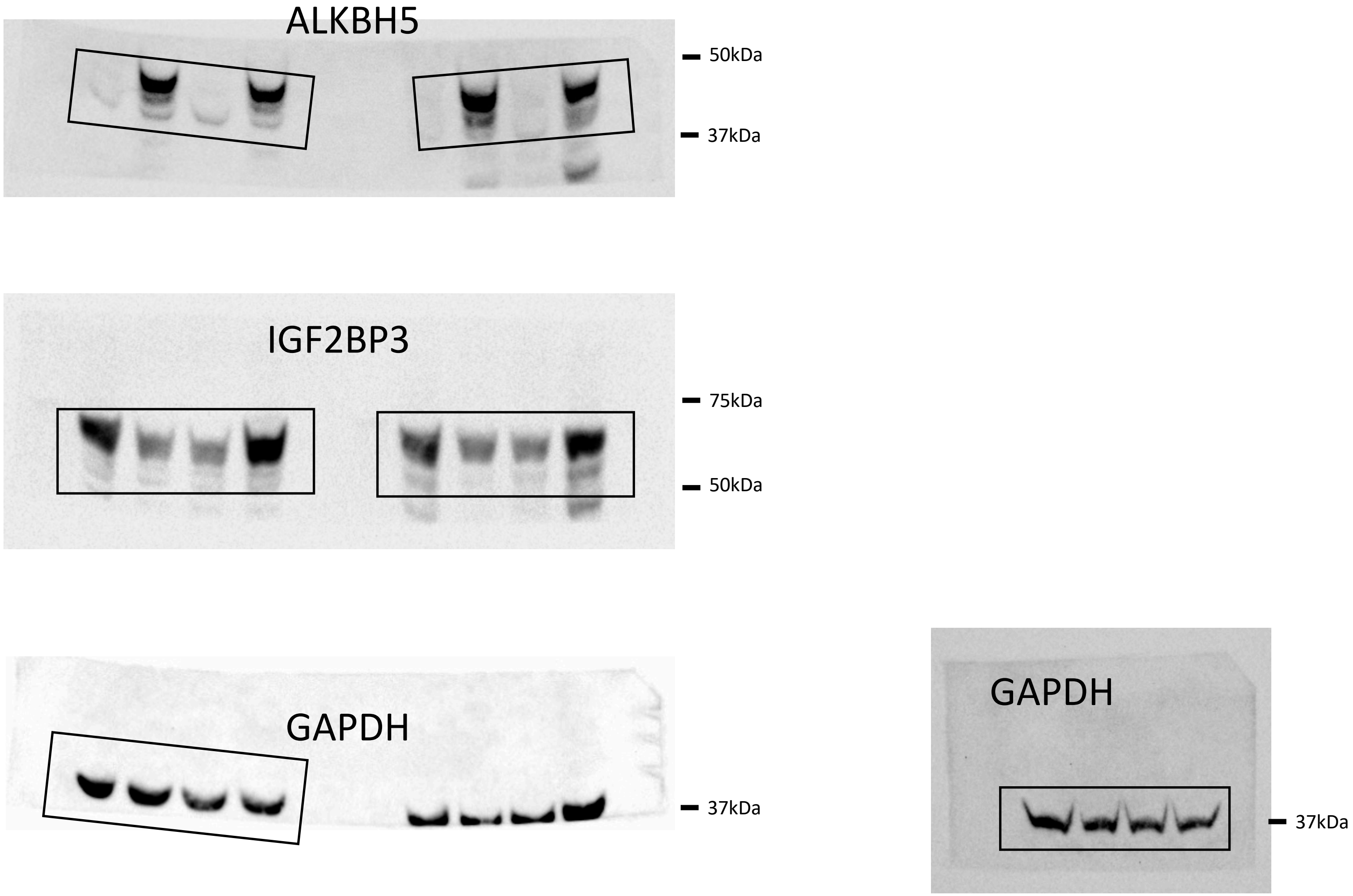

ALKBH5
50kDa
37kDa
IGF2BP3
75kDa
50kDa
GAPDH
37kDa
GAPDH
37kDa

Figure 8D

YTHDF2

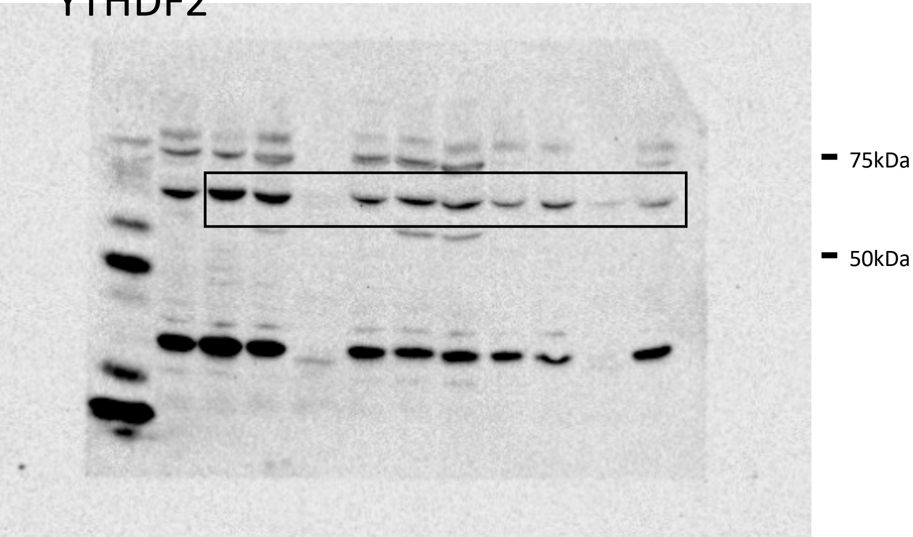

GAPDH

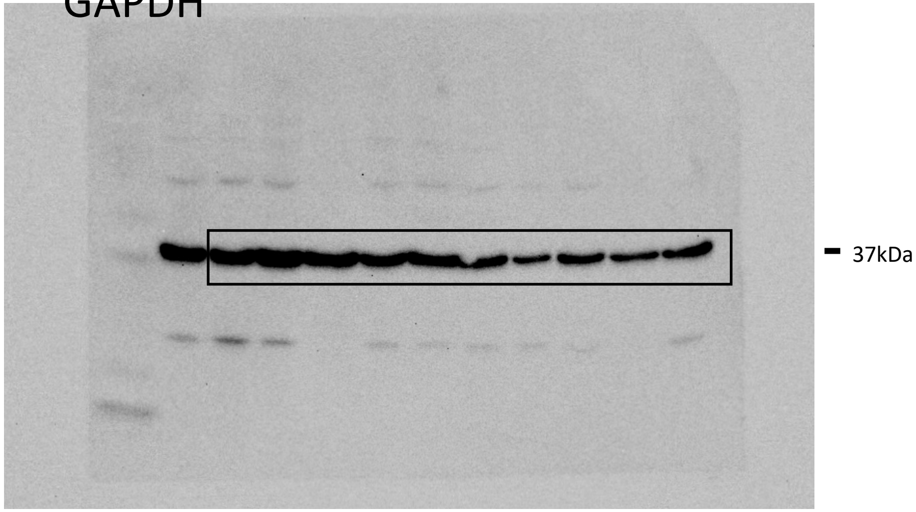

Figure S3D

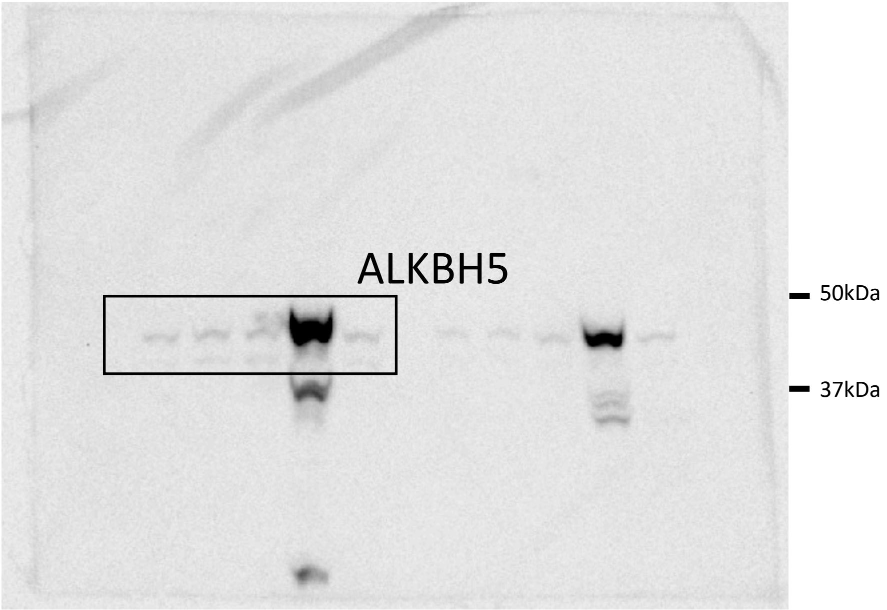

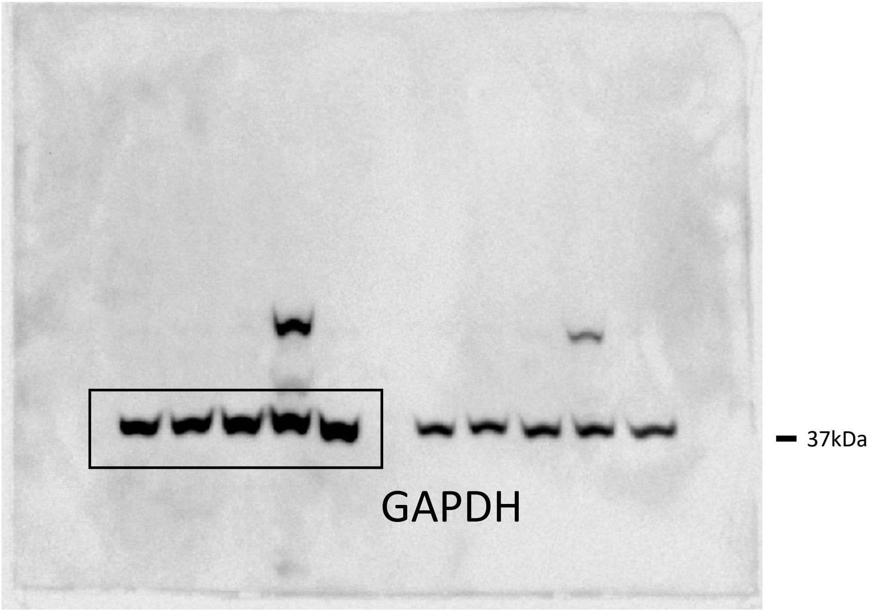

Figure S3E

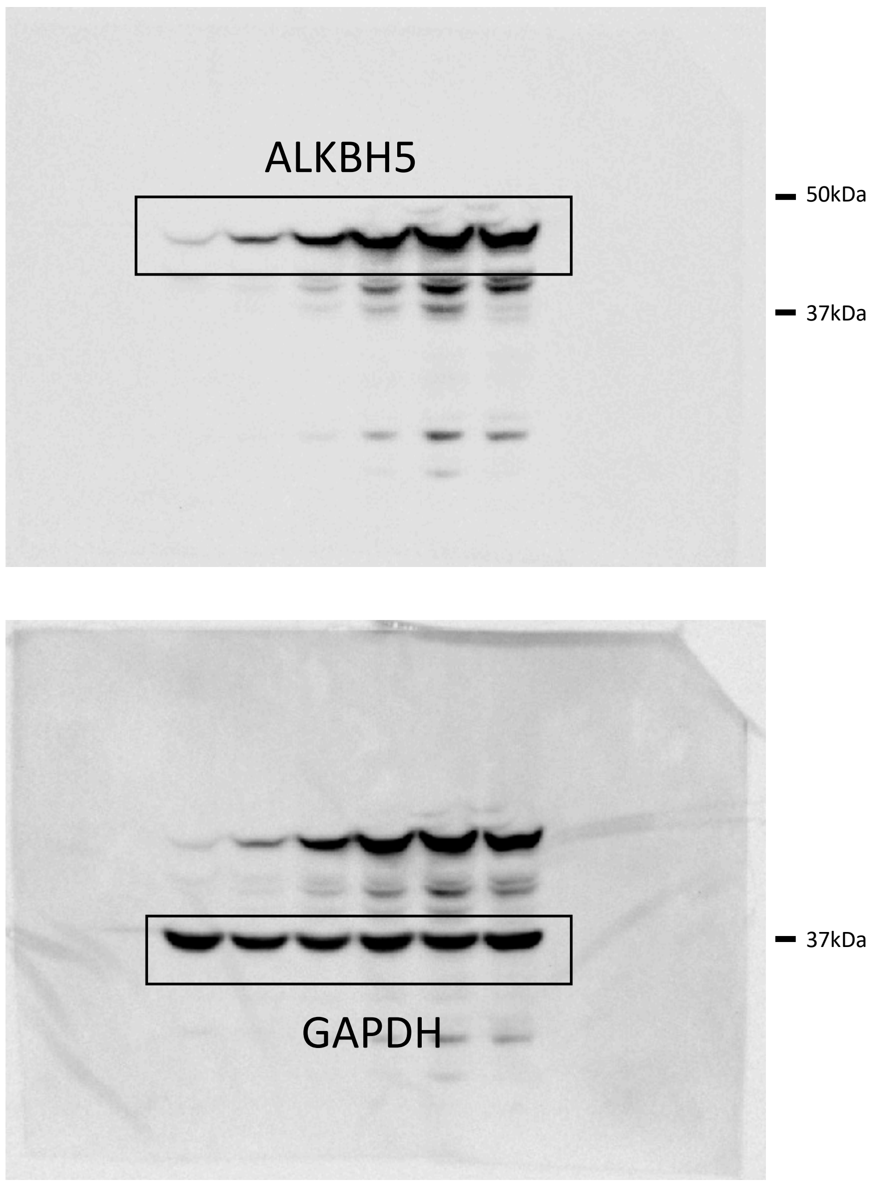

Supplement: Supplementary file 11 — Figure S9 [file 41417_2022_451_MOESM11_ESM.pdf]
